# Anisotropic Interlayer Force Field for Transition Metal Dichalcogenides: The Case of Molybdenum Disulfide

**DOI:** 10.1021/acs.jctc.1c00782

**Published:** 2021-11-01

**Authors:** Wengen Ouyang, Reut Sofer, Xiang Gao, Jan Hermann, Alexandre Tkatchenko, Leeor Kronik, Michael Urbakh, Oded Hod

**Affiliations:** † Department of Engineering Mechanics, School of Civil Engineering, Wuhan University, Wuhan, Hubei 430072, China; ‡ School of Chemistry and The Sackler Center for Computational Molecular and Materials Science, Tel Aviv University, Tel Aviv 6997801, Israel; § Machine Learning Group, TU Berlin, Marchstr. 23, 10587 Berlin, Germany; ∥ Department of Mathematics, FU Berlin, Arnimallee 14, 14195 Berlin, Germany; # Department of Physics and Materials Science, University of Luxembourg, L-1511 Luxembourg City, Luxembourg; ∇ Department of Molecular Chemistry and Materials Science, Weizmann Institute of Science, Rehovoth 76100, Israel

## Abstract

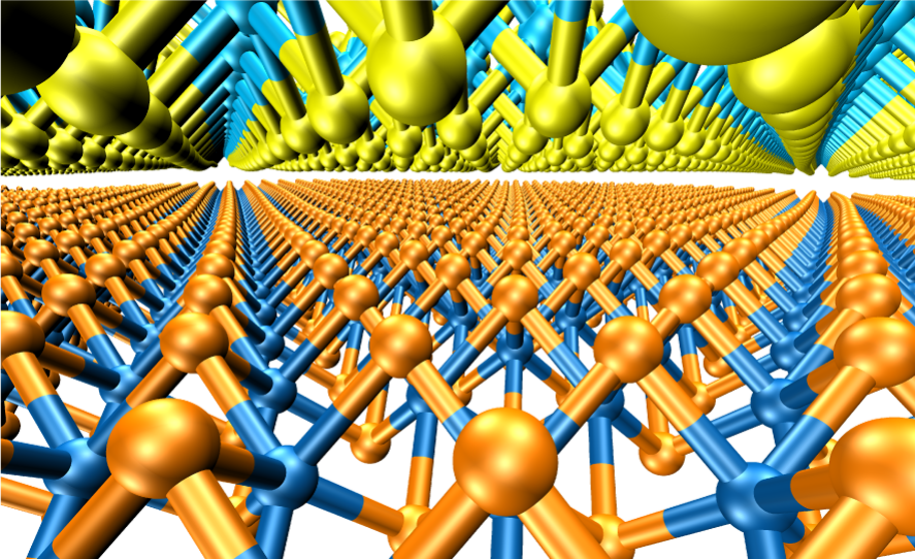

An anisotropic interlayer force field that describes the interlayer interactions in molybdenum disulfide (MoS_2_) is presented. The force field is benchmarked against density functional theory calculations for both bilayer and bulk systems within the Heyd–Scuseria–Ernzerhof hybrid density functional approximation, augmented by a nonlocal many-body dispersion treatment of long-range correlation. The parametrization yields good agreement with the reference calculations of binding energy curves and sliding potential energy surfaces for both bilayer and bulk configurations. Benchmark calculations for the phonon spectra of bulk MoS_2_ provide good agreement with experimental data, and the calculated bulk modulus falls in the lower part of experimentally measured values. This indicates the accuracy of the interlayer force field near equilibrium. Under external pressures up to 20 GPa, the developed force field provides a good description of compression curves. At higher pressures, deviations from experimental data grow, signifying the validity range of the developed force field.

Two-dimensional (2D) transition metal dichalcogenide (TMD) materials have attracted significant attention in recent years due to their unique electrical,^1−5^ optical,^6−8^ thermal,^9−12^ and tribological^13−15^ properties, which are dominated by weak interlayer van der Waals (vdW) interactions and intricate moiré superlattices formed by their heterostructures. Therefore, accurate modeling of interlayer interactions in such layered materials is of paramount importance for obtaining microscopic understanding and a quantitative description of their physical properties. Often, density functional theory (DFT) is the tool of choice for this purpose.^16−19^ Nevertheless, when considering the dynamical phenomena of TMD interfaces of nanoscale dimensions and beyond, the computational burden associated with DFT calculations limits their applicability. In such cases, classical force field based methods may serve as an alternative workhorse, providing a desirable balance between accuracy and efficiency, when appropriately tailored against reliable *ab initio* data for relevant systems.

It is well known that standard isotropic force fields are unsuitable for the simultaneous description of both binding energy (BE) curves and sliding potential surfaces in 2D materials. Kolmogorov and Crespi therefore proposed an alternative approach that separates the treatment of intra- and interlayer interactions, where the latter depends on lateral interatomic distances.^20,21^ Such interlayer potentials (ILP) were used successfully for, e.g., graphitic and hexagonal boron nitride (*h*-BN) based systems,^22−26^ but only recently was such an ILP developed for TMDs and used successfully for capturing structural transformations in their moiré superlattices.^27^

Generally speaking, the quality of an ILP (or indeed of any force field) is at best as good as the reference data it was parameterized against. The abovementioned ILP for TMDs was based on the vdW-DF-C09 nonlocal density functional, which is a popular choice for the description of dispersively bound systems.^28^ An alternative approach to nonlocal density functionals is the use of dispersion-augmented DFT, as given, e.g., by the Tkatchenko–Scheffler (TS) method^19,29−32^ and its many-body dispersion (MBD) extension.^33,34^ When used in conjunction with the Heyd–Scuseria–Ernzerhof (HSE) hybrid density functional approximation,^35−37^ this approach was found to provide reliable equilibrium distances, binding energies,^19,29,30,38^ and elastic constants,^19,30^ for layered materials of weak polarizability or ionic character. Based on this methodology, the Kolmogorov and Crespi approach was recently generalized to generate a registry-dependent ILP^22−25^ for graphitic and *h*-BN systems, which is accurate in both the equilibrium and sub-equilibrium interlayer distance regimes.^25,26^

In light of the above success, it is of interest to extend our registry-dependent ILPs to TMDs. However, because TMDs are highly polarizable, the situation becomes more involved as the conventional MBD treatment fails.^39−41^ To address this issue, we adopt the newly developed nonlocal many-body dispersion approach (MBD-NL),^18^ which substantially improves the description of ionic systems and polarizable materials, such as TMDs, by including the Vydrov and Van Voorhis (VV) polarizability functional.^42^ Here, we explore the use of MBD-NL for generating reference data, against which our ILP is parametrized, and find that this approach yields accurate results.

Before considering TMDs, we compare the results of the MBD and MBD-NL approaches for the binding energies of the less polarizable graphene and *h*-BN interfaces [see Section S1 of the Supporting Information (SI) for further details].^25,26^ For bilayer graphene and bulk graphite, the binding energies obtained using HSE + MBD-NL are found to be 20.39 and 45.46 meV/atom, respectively, which are ∼17 and ∼15% lower than the corresponding values calculated using HSE + MBD (24.67 and 53.29 meV/atom, see Table S1 in the SI). Similarly, for bilayer and bulk *h*-BN, the HSE + MBD-NL binding energies of 20.31 and 44.77 meV/atom, respectively, are lower by ∼25 and ∼23% than those obtained using HSE + MBD (27.37 and 58.17 meV/atom). Importantly, despite these differences, the interlayer distance as a function of the applied pressure (*c*–*P* curves) calculated by the ILP parameters fitted against both the HSE + MBD and the HSE + MBD-NL reference data are close to each other and agree well with experimental measurements. Accordingly, the bulk moduli extracted from the pressure–volume (*P*–*V*) curves obtained using the HSE + MBD-NL parameterized ILP only slightly deviate (by ∼1–2 and ∼6–8 GPa for graphite and bulk *h*-BN, respectively) from the experimental values (see Section S1.3 of the SI for further details).

Having validated the consistency of the previously used HSE + MBD method and the HSE + MBD-NL approach adopted here for graphene and *h*-BN, we now turn to consider interfaces of molybdenum disulfide (MoS_2_)—a prominent member of the TMD family. We start by performing reference HSE + MBD-NL binding energy (BE) calculations for bilayer and bulk MoS_2_, at interlayer distances in the range of 5.0–15 Å. This range includes the sub-equilibrium interlayer distance regime, which is important for describing the tribological properties of layered materials. We consider five high-symmetry stacking configurations of MoS_2_, three of which are associated with the antiparallel (type I) configuration and two with the parallel (type II) configuration,^2,3,16,17^ as illustrated in Figure 1.

**Figure 1:**
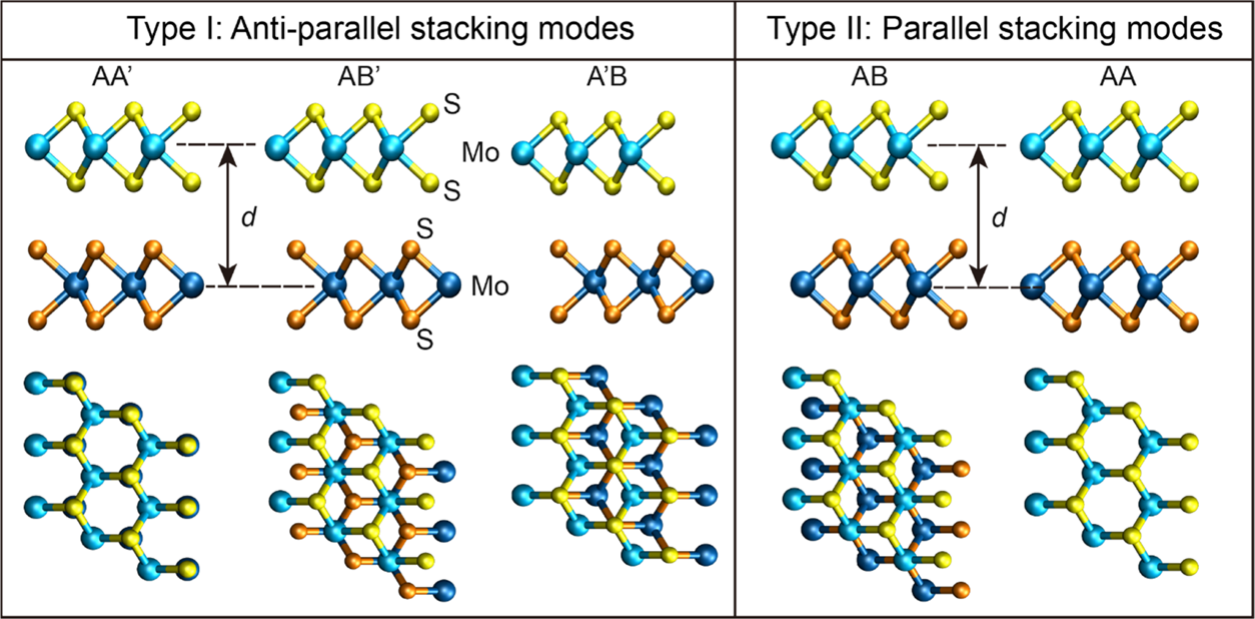
MoS_2_ high-symmetry stacking modes. Left: three high-symmetry stacking modes of the antiparallel configuration. Right: two high-symmetry stacking modes of the parallel configuration. Side and top views of each stacking mode are provided in the upper and lower rows, respectively. The interlayer distance, *d*, is defined as the distance between the Mo planes of adjacent layers. For clarity, atoms residing in different layers are marked with different colors as labeled in the left panel.

The reference DFT data are obtained using the MBD-NL-augmented HSE functional, as implemented in the FHI-AIMS code,^43^ with the tier 2 basis set^44^ using tight convergence settings including all grid divisions and a denser outer grid. Relativistic effects near the nucleus are accounted for by the atomic zero-order regular approximation (ZORA).^43^ For the 2D system, a vacuum size of 100 Å was used with a *k*-grid of 19 × 19 × 1 points. For the three-dimensional (3D) system, a *k*-grid of 19 × 19 × 5 points was used. The five structures shown in Figure 1 were formed by stacking two preoptimized MoS_2_ monolayers. Binding energy curves and sliding energy surfaces were then obtained by rigidly shifting the two layers with respect to each other. Convergence of the DFT results with respect to various calculation parameters is demonstrated in Section S2 in the SI.

Figure 2 presents BE curves calculated for (a) the fully periodic structures of bulk MoS_2_ and (b) the lateral periodic structures of bilayer MoS_2_, at the five high-symmetry stacking modes (open symbols of different colors). As may be expected, both the bilayer and bulk systems possess a similar interlayer distance, where the latter has a BE nearly twice as large as that of the former, due to interlayer interactions of each layer with its two nearest neighboring layers. All HSE + MBD-NL calculations provide bilayer MoS_2_ equilibrium distances and binding energies within 2% and 20% of random phase approximation (RPA) results, respectively, for all stacking modes (see Table 1). We note that the remaining differences may be partly attributed to the approximate nature of the RPA calculation itself.^26^ For reference, we also added the available literature results based on PBE + D2^17^ and vdW-DF-C09^27^ calculations. Importantly, we find that vdW-DF-C09, previously used to fit ILPs for TMDs, overestimates binding energies. This is in agreement with previous findings for graphene on metal surfaces.^45^ HSE + MBD-NL sliding potential energy surfaces (PESs) of bulk and bilayer MoS_2_ at a fixed interlayer distance of 6.2 Å are presented in the left panels of Figures 3 and 4, respectively, for the antiparallel (panel a) and parallel (panel d) configurations.

**Figure 2:**
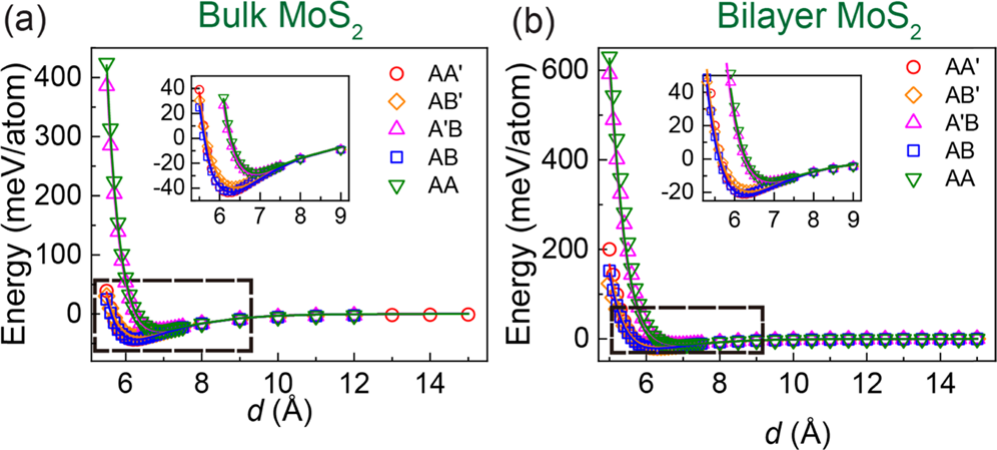
Binding energy curves of the fully periodic structures of bulk MoS_2_ (a) and the laterally periodic structures of bilayer MoS_2_ (b) calculated using HSE + MBD-NL (open symbols), along with the corresponding ILP fits (solid lines). Three stacking modes of the antiparallel configuration (AA′—red, AB′—orange, and A′B—magenta) and two stacking modes of the parallel configuration (AB—blue and AA—green) of MoS_2_ are considered (see Figure 1). The parameters presented in Table S9 in the SI are used to perform the ILP calculations. The reported energies are measured relative to the value obtained for infinitely separated layers and are normalized by the total number of atoms in the unit cell (six atoms). The insets provide zoom-in on the equilibrium interlayer distance region, marked by dashed black rectangles. In the bulk system, *d* represents the distance between all adjacent layers.

**Figure 3:**
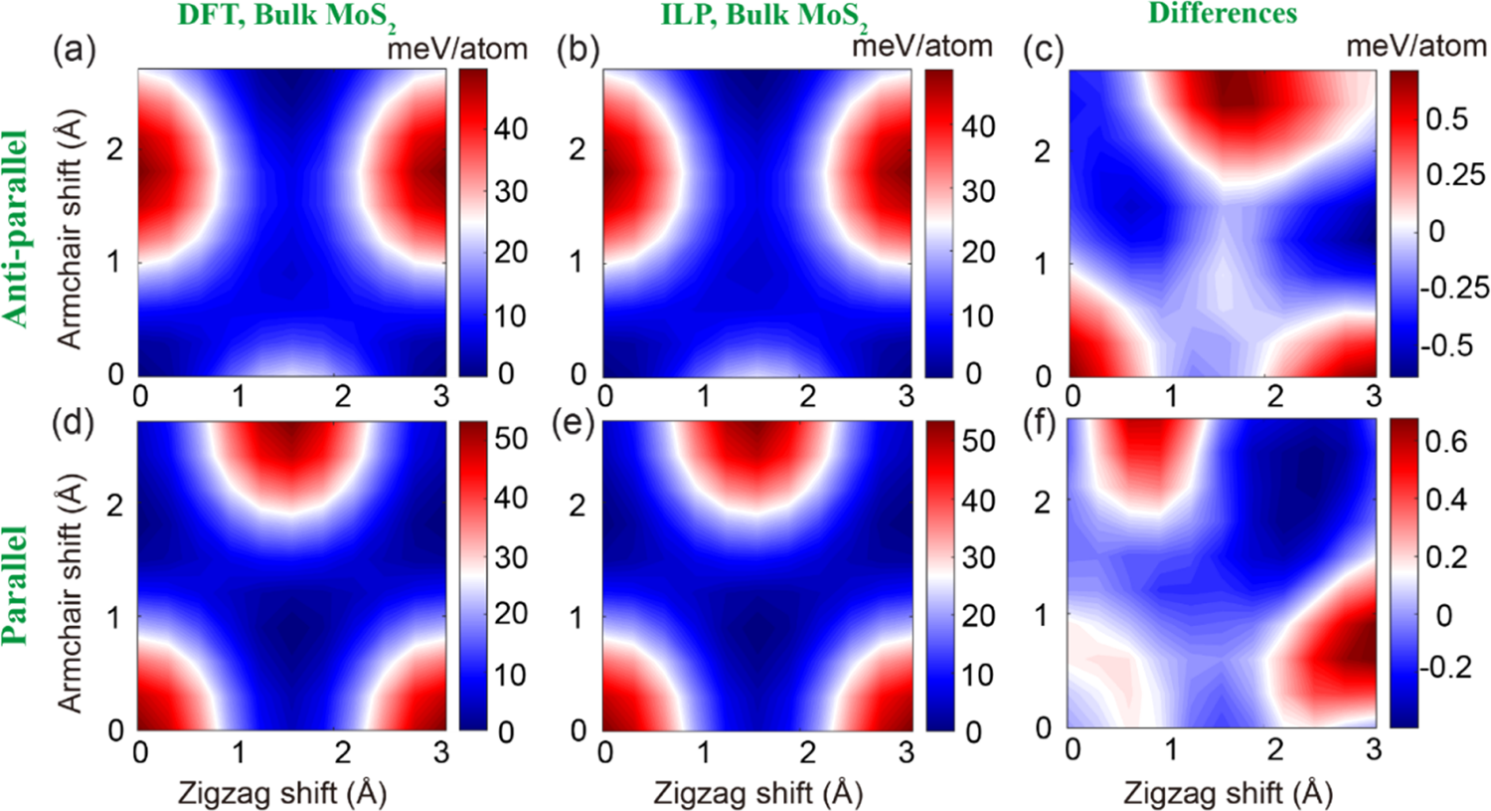
Sliding energy surfaces of bulk MoS_2_, calculated at an interlayer distance of 6.2 Å with periodic boundary conditions applied along both lateral and vertical directions. The first and second rows present the sliding energy surfaces obtained for the antiparallel and parallel configurations, respectively, calculated using (a, d) HSE + MBD-NL and (b, e) the ILP. The differences between the DFT reference data and the ILP results are given in panels (c) and (f). The parameters of Table S9 in the SI are used for the ILP calculations. The reported energies are measured relative to values obtained at the AA′ and AB stacking modes for the antiparallel and parallel configurations, respectively, and are normalized by the total number of atoms in the unit cell (six atoms).

**Figure 4:**
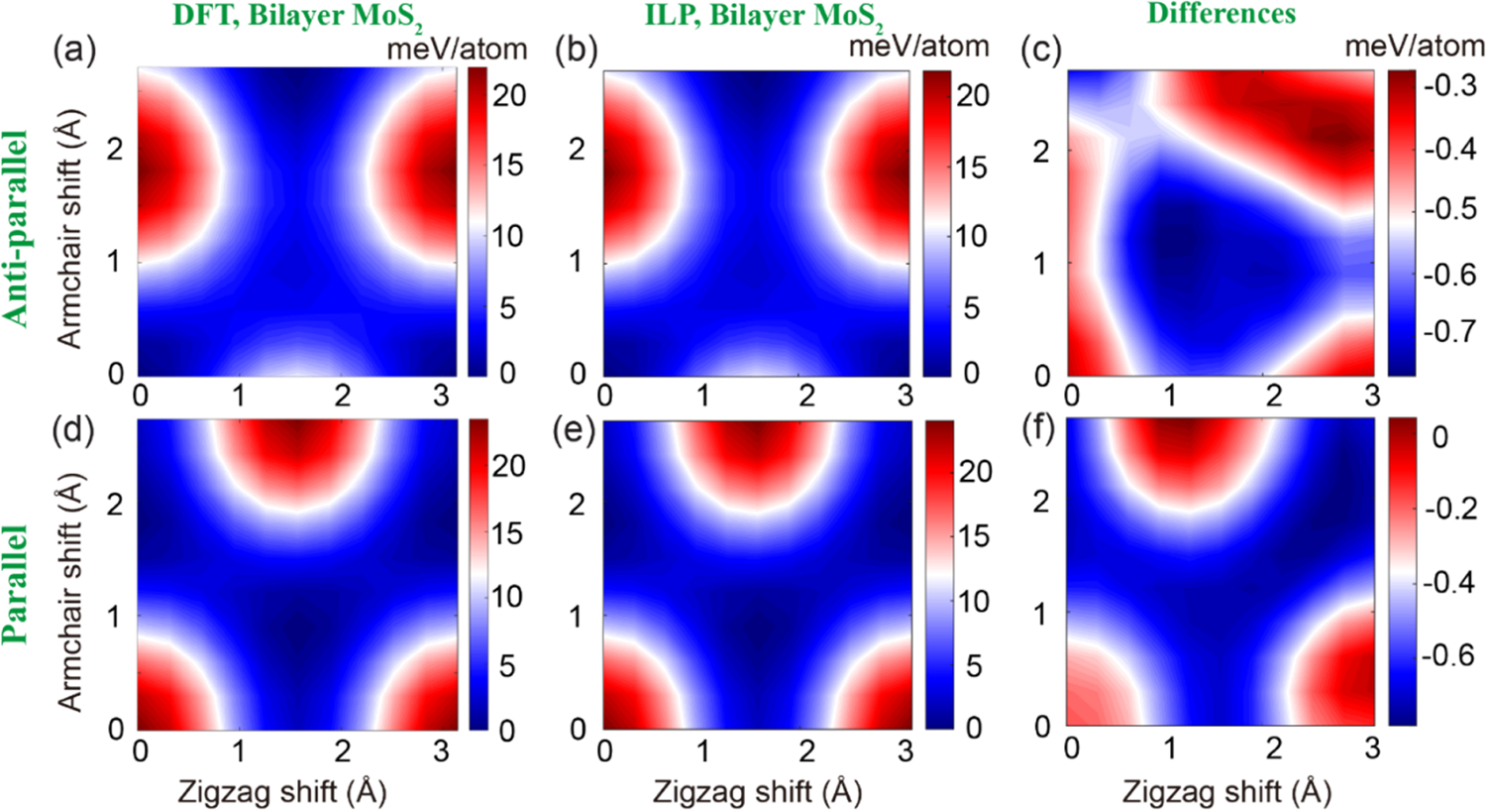
Sliding energy surfaces of bilayer MoS_2_, calculated at an interlayer distance of 6.2 Å with periodic boundary conditions applied along the lateral direction. The first and second rows present the sliding energy surfaces obtained for the antiparallel and parallel configurations, respectively, calculated using (a, d) HSE + MBD-NL and (b, e) the ILP. The differences between the DFT reference data and the ILP results are given in panels (c) and (f). The parameters of Table S9 in the SI are used for the ILP calculations. The reported energies are measured relative to values obtained at the AA′ and AB stacking modes for the antiparallel and parallel configurations, respectively, and are normalized by the total number of atoms in the unit cell (six atoms).

**Table 1:** MoS_2_ Equilibrium Interlayer Distance, *d*_eq_ (Å), and Binding Energy, *E*_b_ (meV/atom), Calculated at Several Stacking Modes Using Various DFT Methods and the ILP^a^

methods		RPA^16^ (bilayer)		PBE + DFT D2^17^ (bilayer)		vdW-DF-C09^27^ (bilayer)		HSE + MBD-NL (bilayer)		HSE + MBD-NL (bulk)		ILP-MBD-NL (bilayer)		ILP-MBD-NL (bulk)	
stacking modes		*d*_eq_	*E*_b_	*d*_eq_	*E*_b_	*d*_eq_	*E*_b_	*d*_eq_	*E*_b_	*d*_eq_	*E*_b_	*d*_eq_	*E*_b_	*d*_eq_	*E*_b_
antiparallel configurations	AA′	6.27	27.1	6.21	25.13	6.0	∼40	6.24	21.1	6.24	43.6	6.29	21.42	6.29	43.29
	AB′	6.26	23.1	6.28	22.98			6.34	18.6	6.33	38.7	6.39	19.56	6.39	39.58
	A′B	6.78	15.1	6.78	16.04			6.86	13.5	6.85	28.2	6. 91	13.57	6.88	28.75
parallel configurations	AB	6.17	25.6	6.21	25.04			6.24	20.6	6.23	42.7	6.29	20.40	6.29	43.20
	AA	6.77	16.0	6.81	15.83	6.7	∼22	6.90	13.3	6.88	27.6	6.95	13.05	6.91	27.91

a An intralayer lattice constant of 3.144 Å is used.

These reference binding energy curves and sliding energy surfaces serve to parameterize the registry-dependent ILP, which is able to describe the strongly anisotropic character of the layered materials under study. To this end, we generalize the ILP functional form previously developed for graphene and *h*-BN systems,^22−26^ to consider the sublayer structure characterizing each TMD layer. Here, the long-range vdW attraction, *V*_att_(*r*_*ij*_), and short-range Pauli repulsion, *V*_rep_(*r*_*ij*_,***n***_*i*_,***n***_*j*_), between any pair of atoms, *i* and *j*, residing in adjacent MoS_2_ layers associated with local normal vectors ***n***_*i*_ and ***n***_*j*_ (see Figure 5), respectively, and separated by a distance *r*_*ij*_, are evaluated using the following pairwise expressions:^22−24^
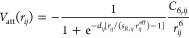
1
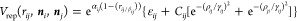
2where *C*_6,*ij*_ is the pairwise dispersion coefficient, *r*_*ij*_^eff^ is the sum of the effective equilibrium vdW atomic radii, and *d*_*ij*_ and *s*_*R*,*ij*_ are unit-less parameters defining the steepness and onset of the short-range Fermi–Dirac type damping function. In eq 2, ε_*ij*_ and *C*_*ij*_ are constants that set the energy scales associated with the isotropic and anisotropic repulsions, respectively, β_*ij*_ and γ_*ij*_ set the corresponding interaction ranges, and α_*ij*_ is a parameter that sets the steepness of the isotropic repulsion function. The lateral interatomic distance ρ_*ij*_(ρ_*ij*_) is defined as the shortest distance from atom *j*(*i*) to the surface normal, ***n***_*i*_(***n***_*j*_), at the position of atom *i*(*j*) (see Figure 5)
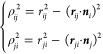
3

**Figure 5:**
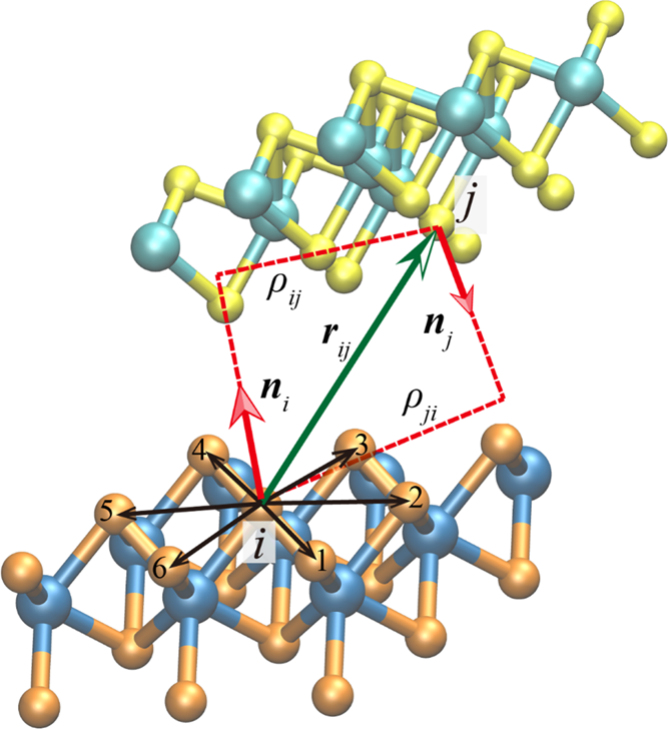
Definition of local normal vectors for MoS_2_. For each atom *i*, its six nearest neighboring atoms within the same sublayer are chosen to define its normal vector ***n***_*i*_. The distance vector and the lateral distances between atoms *i* and *j* residing in adjacent layers are marked by ***r***_*ij*_ (green arrow) and ρ_*ij*_ and ρ_*ji*_ (dashed red lines), respectively. The color scheme is the same as that used in Figure 1, where atoms residing in different layers are marked with different colors.

Since each MoS_2_ layer contains two sublayers of S atoms and one sublayer of Mo atoms (see Figure 1), the definition of the normal vectors used for graphene and *h*-BN is no longer valid for MoS_2_. Thus, we propose a new definition of the surface normal vector of MoS_2_. As illustrated in Figure 5 for the specific case of a sulfur atom, for each atom *i*, its six nearest neighboring atoms belonging to the same sublayer are chosen to define the normal vector ***n***_*i*_, which is calculated as follows:
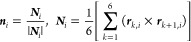
4where ***r***_*k*,*i*_ = ***r***_*k*_ – ***r***_*i*_, *k* = 1, 2,..., 6 and the summation is understood to be cyclic, i.e., ***r***_7,*i*_ = ***r***_1,*i*_. Finally, the total potential is given by the following expression:
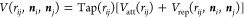
5where
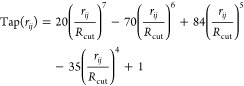
6is a taper function that provides a continuous long-range cutoff (up to the third derivative) that dampens the interactions between any pair of atoms *i* and *j* residing in adjacent layers, at interatomic separations (*r*_*ij*_, see Figure 5) exceeding *R*_cut_ = 16 Å.

The parameters of the interlayer force field are optimized against DFT reference results,^25,26^ which include *M* = *M*_b_ + *M*_s_ data sets (*M*_b_ binding energy curves and *M*_s_ sliding energy surfaces). As detailed above, the binding energy curves are calculated for five high-symmetry stacking modes (see Figure 1), which are denoted by *E*_*m*_^b^(**ξ**), *m* ∈ [1, *M*_b_ = 5]. Similarly, the sliding PESs are denoted by *E*_*m*_^s^(**ξ**), *m* ∈ [1, *M*_s_ = 2]. Here, **ξ** represents the set of potential parameters. Each BE curve and sliding PES contain 26 and 110 data points, respectively. Since the corrugation of the sliding PES is relatively small compared to the BE in the sub-equilibrium regime and we focus on providing a good description of it, the objective function weights for the BE curves and for the sliding PES are set as follows: *w*_*m*_^b^ (*d* < *d*_eq_^*m*^) = 1, *w*_*m*_^b^ (*d* ≥ *d*_eq_^*m*^) = 20, and *w*_*m*_^s^ = 50, where *d*_eq_^*m*^ is the equilibrium interlayer distance for the *m*^th^ stacking mode (see Figure 1).^46^ Parameter optimization is performed by minimizing the following objective function that quantifies the difference between the interlayer potential predictions and the DFT reference data:
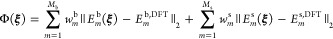
7where ∥···∥_2_ is the Euclidean norm (two-norm) that measures the difference between the ILP predictions and the DFT reference data. Since DFT provides the total energy of the system due to both intralayer and interlayer interactions, it is necessary to extract the interlayer contributions when constructing the reference data. This is achieved by subtracting the total energy of the individual layers from that of the bulk or bilayer system. The parameter optimization was carried out using MATLAB software with an interior-point algorithm.^47,48^ More details of this procedure can be found in refs (25) and (26). The fitting was performed against the bulk reference data with the bilayer DFT reference data serving to benchmark the results. Fitted parameters and benchmark tests are given in Sections S3 and S4 in the SI.

Notably, the ILP BE curves fit well the DFT reference data over the entire interlayer separation range considered (including the sub-equilibrium regime) for all five stacking modes of both the bulk and bilayer systems (see Figure 2 and Table 1). Furthermore, the ILP sliding energy surfaces (see Figures 3b,e and 4b,e) match well the reference DFT data with a maximal deviation smaller than 1.3 and 3.4% of the overall PES corrugation for bulk and bilayer MoS_2_, respectively (see Figures 3c,f and 4c,f).

As a benchmark test for the developed MoS_2_ ILP, we computed the phonon dispersion curves of bulk MoS_2_ at zero pressure and temperature, based on diagonalization of the dynamical matrix in LAMMPS, and compared them with experimental data.^49^ To that end, a supercell containing 25 × 25 × 6 unit cells (45 000 atoms) and 201 *q* points was used and a step size of 10^–6^ Å was used for numerical differentiation. Computing the phonon spectrum of bulk MoS_2_ using HSE + MBD-NL turned out to be computationally prohibitive. However, the excellent agreement between ILP and HSE + MBD-NL binding energy curves and sliding energy surfaces allowed us to perform the calculations with the ILP instead. Along with the MoS_2_ ILP, two types of intralayer MoS_2_ force fields, the second-generation reactive empirical bond order (REBO) potential^50,51^ and the Stillinger–Weber (SW) potential,^52^ were used to describe the interactions between atoms within each MoS_2_ layer. The results, highlighted by the green rectangle in Figure 6a,b, show that the dispersion of the low-energy out-of-plane branches (near the Γ point), which are related to the soft flexural modes of the layers, is well described by the ILP (see Figure 6c,d). The larger deviations from the experimental data, observed for the high-energy modes, are mainly caused by the intralayer potential terms, where the REBO and SW potentials give similar behavior for both single-layer and bulk MoS_2_ (see Figure S10 in Section S4 of the SI). Notably, the isotropic Lennard-Jones (LJ) interlayer potential considerably underestimates the out-of-plane phonon energies (see Figure S11 in Section S4 in the SI). More details regarding the corresponding phonon spectra obtained for graphite and bulk *h*-BN systems can be found in Section S1.4 in the SI and refs (53) and (54).

**Figure 6:**
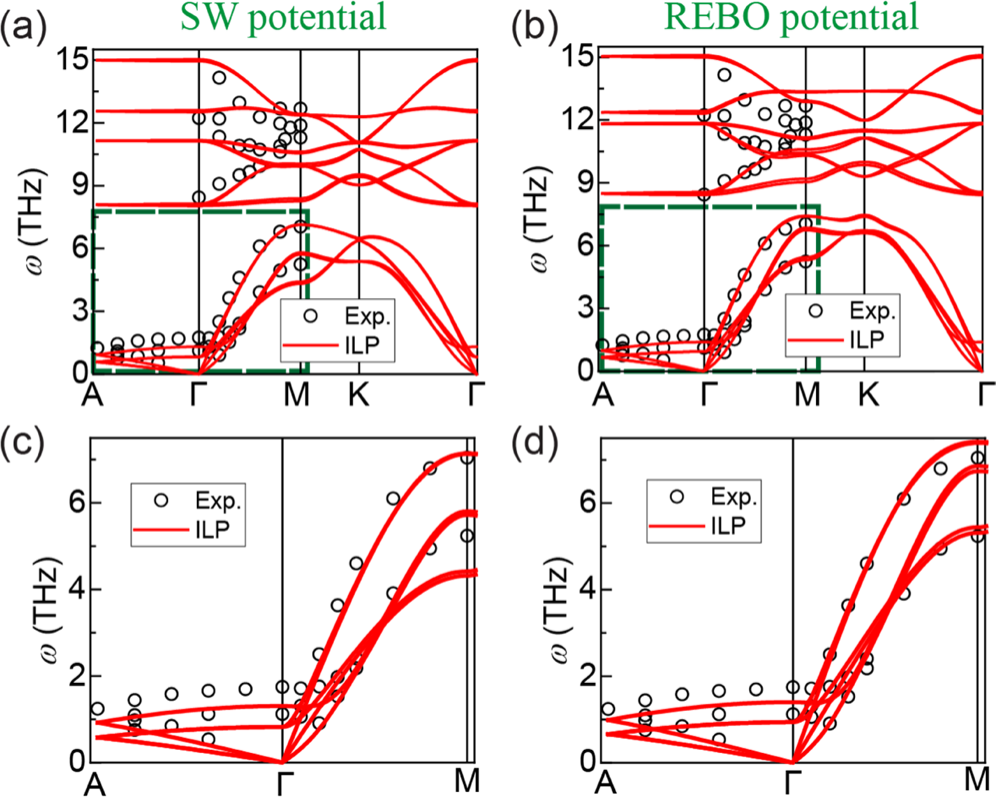
(a) Phonon spectra of bulk MoS_2_ calculated using the ILP with the (a) SW^52^ and (b) REBO^50,51^ intralayer potentials. Red solid lines are dispersion curves calculated using the parameters listed in Table S1. Experimental results of bulk MoS_2_ are presented by open black circles.^49^ Panels (c) and (d) show zoom-ins on the low-energy phonon modes around the Γ point (dashed green rectangles in panels (a) and (b)) for the SW potential and REBO potential, respectively.

To further evaluate the performance of the developed MoS_2_ ILP under hydrostatic pressure, *P*, we calculated the *a*–*P, c*–*P*, and *V*–*P* curves of bulk MoS_2_ describing the dependence of the structural parameters of the solid (*a* and *c* lattice parameters and the volume, *V*, respectively) on the external pressure. To this end, we adopted supercell models consisting of 12 rectangular layers (7.9 nm × 13.7 nm), each containing 1250 molybdenum + 2500 sulfur atoms. The layers were arranged in alternating AA′, AB′, or AB stacking modes (see Figure 1), with a period *c*, initially set equal to 12.42 Å. The second-generation REBO potential^50,51^ was used to describe the intralayer interactions within each MoS_2_ layer. Interlayer interactions were modeled using the bulk MoS_2_ ILP parameterizations. All MD simulations were performed using the LAMMPS simulation package.^55^ A velocity Verlet integrator with a time step of 1 fs was used to propagate the equations of motion while enforcing periodic boundary conditions in the lateral and vertical directions. A Nosé–Hoover thermostat with a time constant of 0.25 ps was used for constant temperature simulations. To maintain a specified hydrostatic pressure, the three translational vectors of the simulation cell were adjusted independently by a Nosé–Hoover barostat with a time constant of 1.0 ps.^56,57^ To generate the *c*–*P* curves, we first equilibrated the systems in the NPT ensemble at a temperature of *T* = 300 K and a fixed target pressure for 200 ps. After equilibration, the *c* lattice parameter was computed by averaging over a subsequent simulation period of 200 ps. By applying this procedure at different external pressures, ranging from 0 to 55 GPa, the *c*–*P*, *a*–*P*, and *V*–*P* curves were constructed. The comparison of the ILP results with experimental data is shown in Figure 7.

**Figure 7:**
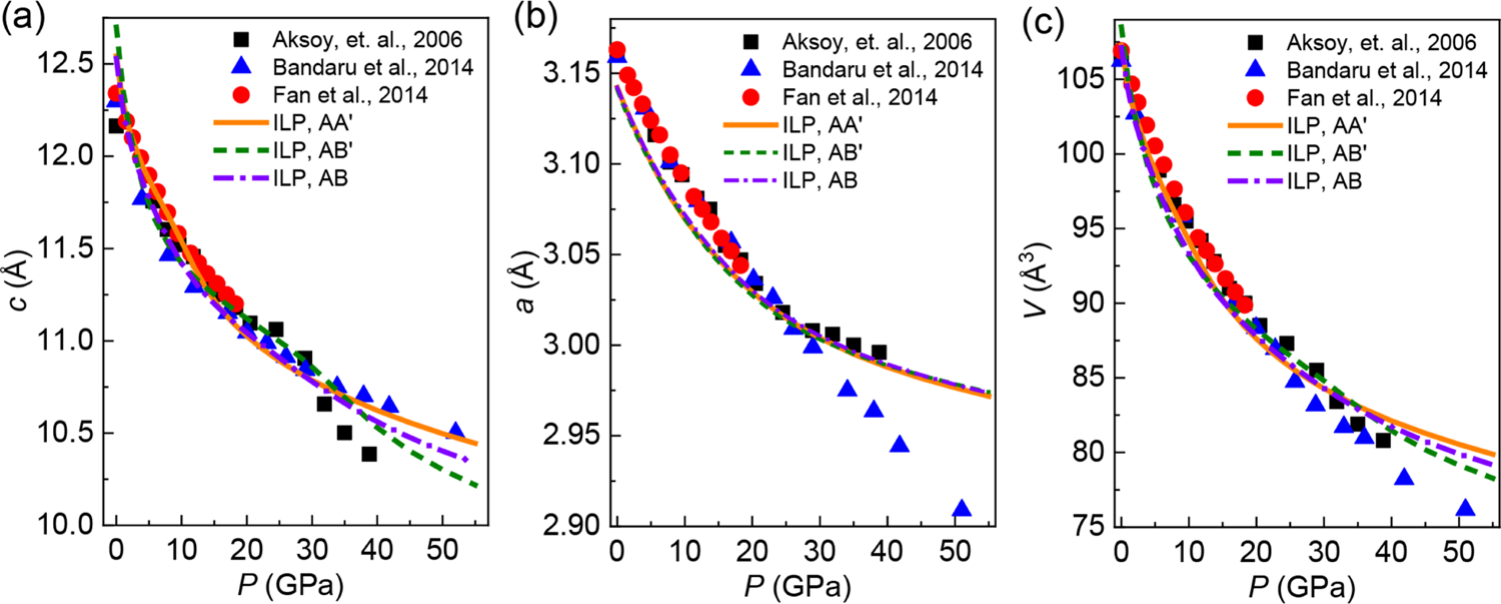
Pressure dependence of the *c* lattice parameter (a), *a* lattice parameter (b), and the unit cell volume (c) of bulk MoS_2_. Experimental results for bulk MoS_2_ are represented by full symbols (black rectangles, blue triangles, and red circles). The NPT simulation results obtained for bulk MoS_2_ at different high-symmetry stacking modes are given by the orange solid (AA′ stacking), green dashed (AB′ stacking), and violet dashed-dotted (AB stacking) lines. Error bars for the simulated data, obtained from the temporal standard deviation of the interlayer distance thermal fluctuations at equilibrium, are smaller than the symbol widths.

Up to a pressure of 20 GPa, good agreement between the calculated results at the optimal AA′ stacking mode (solid red line) and the experimental values (full symbols) is obtained with deviations up to 3.1, 0.66, and 0.95%, for the *c*–*P*, *a*–*P*, and *V*–*P* curves, respectively, at the low-pressure regime (<4 GPa), which decrease with increasing pressure. Notably, in this pressure range, similar pressure dependence is obtained for other stacking modes as well (see full black and blue lines). Above 20 GPa, the deviation of the calculated results from the experimental values generally grows and becomes significant, especially for the *a*–*P* curve. We attribute the increased deviations between the experimental and calculated values in the high-pressure regime to the following points: (i) the reliability of the DFT reference data in the deep sub-equilibrium regime may be compromised due to electronic correlation effects; (ii) the intralayer potential for MoS_2_ might not be accurate under high hydrostatic pressure since it is benchmarked with the properties of MoS_2_ near equilibrium; and (iii) a possible structural phase transition occurring experimentally at a pressure of ∼20 GPa.^58−60^ We note that we did not observe any such phase transition during our simulations, possibly due to their inherently limited time scale.

Finally, as an additional demonstration of the performance of the MoS_2_ ILP, we compared the calculated MoS_2_ bulk moduli with experimental values. The computed bulk moduli were obtained by fitting our calculated *V*–*P* curves (see Figure 7c) to the Murnaghan equation of state (EOS)^61,62^
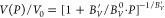
8

Here, *V*_0_ and *V*(*P*) are the unit cell volumes in the absence and presence of external hydrostatic pressure, and *B*_*V*_^0^ and *B*_*V*_^′^ are the bulk modulus and its pressure derivative at zero pressure, respectively. For completeness, we fitted the calculated *V*–*P* curves to two other commonly used equations of state: (i) the Birch–Murnaghan equation^63,64^ and (ii) the Vinet equation,^65,66^ which differ in their description of the dependence of *B*_*V*_ on the pressure, by assuming that it is polynomial and exponential rather than linear as in the Murnaghan EOS (see Section S1.3 of the SI for further details).

As can be seen in Table 2, the experimental values of the bulk modulus and its pressure derivative for bulk MoS_2_ are in ranges of 47.65–70 GPa and 4.5–10.58, respectively. The corresponding ILP results for the bulk modulus fall in the lower part of this range (40–47 GPa) and show relatively weak dependence on the choice of EOS. This corresponds well with the fact that most of the available DFT data fall in the range of 19–46 GPa. The ILP pressure derivative of the bulk modulus falls well within the experimental range, as well. Satisfactory agreement with the experimental values of the intra- and interlayer lattice constants is also achieved for the MoS_2_ ILP and all DFT methods listed in Table 2. The accuracy of the force field and DFT predictions for the intra- and interlayer lattice constants are found to be ∼0.02 and ∼0.5 Å, respectively, apart from the PBE, PW91, and vdW-DF1 DFT results that overestimate both the intra- and interlayer lattice constants. The binding energies obtained using the MBD-NL parameterized ILP are on par with the values reported in several vdW-DF calculations.

**Table 2:** Bulk Modulus (*B*_*V*_) and Its Zero-Pressure Derivative (*B*_*V*_^′^), Intra- (*a*_0_) and Interlayer (*c*_0_) Lattice Constants, and Binding Energies (*E*_bind_) of AA′-Stacked Bulk MoS_2_ Calculated Using the ILP and Compared to the Reported Experimental and First-Principles Values

	methods		*B*_*V*_^0^ (GPa)	*B*_*V*_^′^	*a*_0_ (Å)	*c*_0_ (Å)
experiments	X-ray diffraction^58^		53.4 ± 1^a^	9.2 ± 0.4^a^		
	X-ray diffraction^59^		70 ± 5^a^	4.5^a^	3.159(8)	12.298(3)
	X-ray diffraction^60^		47.65 ± 0.30^a^	10.58 ± 0.08^a^		
	X-ray diffraction^68^		69 ± 2^a^	4.7 ± 0.2^a^	3.163(4)	12.341(4)
first-principles	LDA^69^		41.1^b^		3.13	12.06
	PBE^69^		1.8		3.19	14.01
	PBE^70^		63.36		3.199	12.493
	PBE + D2^69^		46.3		3.20	12.42
	PBESOL^69^		19.2		3.15	12.57
	PW91^69^		1.4		3.21	14.37
	PW91-D2^69^		46.3		3.22	12.39
	vdW-DF1^69^		24.3		3.24	12.96
	vdW-DF2-C09^69^		40.9		3.16	12.26
	vdW-DF2-CX^69^		37.6		3.16	12.27
	vdW-DF2-B86R^69^		37.6		3.18	12.37
	PBE + D3^71^		55^c^		3.16	12.31
	HSE + D2^72^		62.4^c^			
MD simulations^d^	ILP	Murnaghan EOS, eq 1	47 ± 5	7.0 ± 0.7	3.1422(1)	12.5436(4)
		Birch–Murnaghan EOS, eq 2	40 ± 5	11 ± 2		
		Vinet EOS, eq 3	43 ± 3	9.1 ± 0.6		

a Fit with eq 2.

b Fit with eq 1.

c Calculated from elastic constants.

d The MD simulations were performed at 300 K.

The benchmark evaluations presented above demonstrate the validity range of the developed ILP for layered MoS_2_ systems. The force field parameterization against reference calculations based on screened hybrid DFT augmented by nonlocal many-body dispersion corrections yields good agreement with experimental interlayer phonon spectra. The calculated bulk modulus falls within the lower bound of the experimental range. Under external pressures up to 20 GPa, the developed ILP provides a good description of the compression curves. At higher pressures (up to 55 GPa), the deviations between the experimental data and ILP predictions grow to ∼4%.

We note that the HSE + MBD-NL computed MoS_2_ binding energies, PES corrugations, phonon spectra, and bulk modulus are all underestimated or are at the lower end of the range of computational and experimental reference values. We emphasize that this underestimation is not a general feature of the DFT + MBD-NL approach. For example, for AB-stacked bilayer graphene, PBE + MBD-NL yields a binding energy of 17.8 meV/atom (see Table S2 in the SI), which is in excellent agreement with a diffusion Monte Carlo (DMC) value of 17.7 meV/atom,^67^ with the HSE + MBD-NL value being even higher (20.4 meV/atom). A similar conclusion is obtained by considering the calculated phonon spectra and bulk moduli of graphite and *h*-BN (see Figure S7 and Table S8 in the SI). The reasons for the slight but apparently consistent underestimation for MoS_2_ are presently unknown and are outside the scope of this article.

The successful construction of a registry-dependent interlayer potential based on state-of-the-art many-body dispersion-corrected DFT reference data for layered molybdenum disulfide that includes an intricate sublayer structure opens the way for the efficient and accurate simulation of large-scale homogeneous and heterogeneous interfaces based on the vast family of transition metal dichalcogenide layered materials.

## Supporting Information Available

MBD-NL parameterization for graphene and *h*-BN interfaces; convergence tests of the reference DFT calculations; interlayer potential parameters for bulk MoS_2_; and the effect of intra- and interlayer potentials on the calculated phonon spectra of MoS_2_ (PDF)
