# Supplementary material for: Anisotropic Interlayer Force Field for Transition Metal Dichalcogenides: The Case of Molybdenum Disulfide
Source: J Chem Theory Comput. 2021 Nov 1;17(11):7237–45. doi: 10.1021/acs.jctc.1c00782 (PMC8592503; doi:10.1021/acs.jctc.1c00782)
Supplement: Supplementary file 1 — ct1c00782_si_001.pdf [file ct1c00782_si_001.pdf]

Supporting Information for "Anisotropic Interlayer Force Field for Transition Metal Dichalcogenides: The Case of Molybdenum Disulfide"

Wengen Ouyang,[1#] Reut Softer,[2#] Xiang Gao,[2#] Jan Hermann,[3,4] Alexandre Tkatchenko,[5,6] Leeor Kronik,[7] Michael Urbakh,[2*] and Oded Hod[2]

[1]*Department of Engineering Mechanics, School of Civil Engineering, Wuhan University, Wuhan, Hubei 430072, China*

[2]*School of Chemistry and The Sackler Center for Computational Molecular and Materials Science, Tel Aviv University, Tel Aviv 6997801, Israel*

[3] *TU Berlin, Machine Learning Group, Marchstr. 23, 10587 Berlin, Germany*

[4] *FU Berlin, Department of Physics, Arnimallee 14, 14195 Berlin, Germany*

[5]*Fritz-Haber-Institut der Max-Planck-Gesellschaft, Faradayweg 4-6, D-14195 Berlin, Germany*

[6]*Physics and Materials Science Research Unit, University of Luxembourg, L-1511, Luxembourg*

[7]*Department of Molecular Chemistry and Materials Science, Weizmann Institute of Science, Rehovoth 76100, Israel*

#: These authors contribute equally to this work.

Corresponding author: urbakh@tauex.tau.ac.il

This supporting information document includes the following sections:

1. MBD-NL Parameterization for Graphene and *h*-BN Interfaces.
2. Convergence Tests of the Reference DFT Calculations.
3. Interlayer Potential Parameters for bulk MoS_2.
4. Effect of Intra- and Inter-Layer Potentials on the Calculated Phonon Spectra of MoS_2.

1. MBD-NL Parameterization for Graphene and *h*-BN Interfaces

For completeness, we repeat the MBD-NL parameterization for our previously developed ILP for graphene and *h*-BN interfaces.[11, 12] Similar to the MoS_2 calculations, the DFT calculations are performed using the HSE06 functional augmented by the MBD-NL correction using the FHI-AIMS code,[18] with the tier-2 basis-set[19] and tight convergence settings including all grid divisions and a denser outer grid. Relativistic effects are neglected for carbon, boron, and nitride atoms. For the 2D periodic system, a vacuum size of 50 Å was used with a *k*-grid of 19×19×1 points. For the three-dimensional (3D) periodic system, a *k*-grid of 19×19×7 points was used. Using these settings, the binding energy is converged to within ~0.4 meV/atom for bilayer graphene and bilayer *h*-BN/graphene, and ~0.05 meV/atom for the other bilayer and bulk configurations considered, as shown in **Figure S9** in Sec. 2. The different structures appearing in **Figure S1** are formed by stacking two monolayers that were pre-optimized using the HSE functional and the split-valence double-ζ 6-31G** Gaussian basis-set,[20] as implemented in the GAUSSIAN suite of programs.[21] Binding energy curves and sliding energy surfaces are then obtained by rigidly shifting the two layers with respect to each other.

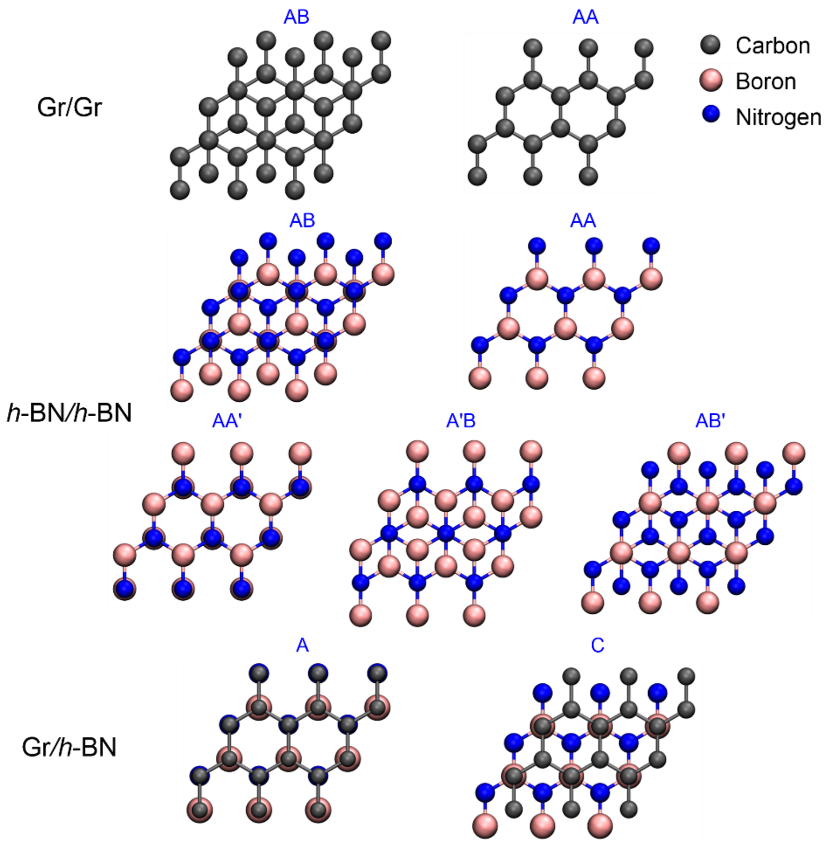

Figure S1. *High symmetry stacking modes of the graphene and h-BN homo- and hetero-structures considered herein. The gray, mauve and blue spheres represent carbon, boron and nitrogen atoms, respectively.*

1.1. Binding energy curves

Figure S2 presents binding energy curves obtained using HSE+MBD-NL calculations (open symbols) for bulk (left column) and bilayer (right column) graphene (top panels), *h*-BN (middle panels), and their heterostructures (lower panels), at interlayer distances in the range of 2-10 Å and 2-15 Å, respectively. ILP fitting results are presented by solid lines.

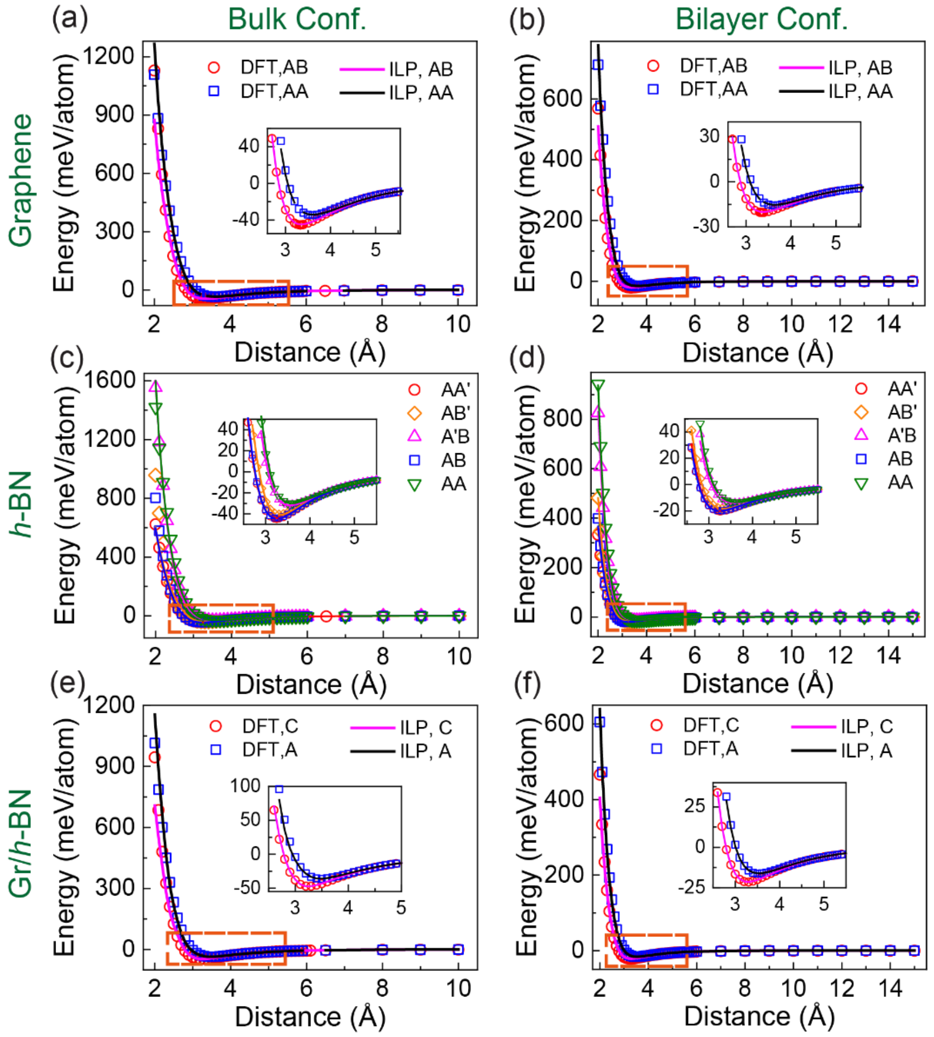

Figure S2. *Binding energy curves of the periodic structures of graphene (upper panels), h-BN (middle panels), and alternating graphene/h-BN heterojunctions (bottom panels), calculated at the HSE+MBD-NL level of theory for bulk (left column, open symbols) and bilayer (right column, open symbols) systems at various stacking modes, along with the corresponding ILP fits (solid lines). The reported energies are measured relative to the value obtained for infinitely separated layers and are normalized by the total number of atoms in the unit-cell (4 atoms). The insets provide a zoom-in on the equilibrium interlayer distance region.*

Notably, the ILP can be well fitted (using the procedure described in Refs. 11, 12) against both bulk and bilayer reference HSE+MBD-NL binding energy data throughout the entire interlayer distance range considered, which extends deep into the sub-equilibrium regime.

A comparison of binding energies obtained using HSE+MBD and HSE+MBD-NL is given in **Table S1**, from which we find that the binding energies predicted by HSE+MBD-NL are lower by 15%-25% compared to those calculated by HSE+MBD, whereas the differences between the equilibrium interlayer distances obtained using the two methods are within 2%.

Table S1. Equilibrium interlayer distance (d_{eq}, Å), binding energy (E_b, meV/atom) and maximum sliding energy corrugation (U_{corr}, meV/atom) calculated using the HSE+MBD and HSE+MBD-NL DFT methods for bilayer and bulk graphene and *h*-BN based systems. The values in the brackets show the relative difference between HSE+MBD-NL and HSE+MBD DFT data.

Methods	HSE+MBD[11] (Bilayer)			HSE+MBD-NL (Bilayer)			HSE+MBD[12] (Bulk)			HSE+MBD-NL (Bulk)		
Systems	d_{eq}	E_b	U_{corr}	d_{eq}	E_b	U_{corr}	d_{eq}	E_b	U_{corr}	d_{eq}	E_b	U_{corr}
Graphene (AB stacked)	3.4	24.67	6.71[a]	3.36 (-1.1%)	20.39 (-17%)	6.50[a] (-3.1%)	3.3	53.29	18.06[b]	3.33 (0.9%)	45.46 (-15%)	17.42[b] (-3.5%)
h-BN (AA' stacked)	3.3	27.37	8.97[b]	3.26 (-1.2%)	20.31 (-25%)	9.01 (0.45%)	3.3	58.17	18.01[b]	3.24 (-1.8%)	44.77 (-23%)	18.12[b] (0.61%)
Graphene/*h*-BN (C-stacked)	3.3	27.14	7.29[b]	3.30 (0.0%)	21.40 (-21%)	7.07 (-3.0%)	3.3	58.00	14.37[b]	3.30 (0.0%)	47.05 (-19%)	13.76[b] (-4.2%)

[a]calculated at d = 3.4 Å, [b]calculated at d = 3.3 Å.

Having compared the HSE06+MBD-NL and HSE06+MBD results for graphene and *h*-BN, we further evaluate the performance of our reference HSE06+MBD-NL calculations against high-accuracy computational approaches including diffusion Monte Carlo (DMC), quantum Monte Carlo (QMC), and random phase approximation (RPA) calculations, as well as available experimental data. **Table S2** compares the binding energy of bilayer graphene obtained using DMC, PBE+MBD, PBE+MBD-NL, HSE+MBD, and HSE+MBD-NL results. The differences between the various MBD results and the DMC value are all within 7 meV/atom, where the MBD-NL calculations provide better agreement to within 3 meV/atom. We note that these differences may be partly attributed to the fact that the DMC and DFT calculations are performed at somewhat different interlayer distances. The PBE+MBD-NL result seems to be in better agreement with the DMC value than the HSE+MBD-NL result, but these differences are marginal given the accuracy uncertainty of our calculations.

Table S2. Binding energy (BE) of bilayer graphene obtained using DMC, PBE+MBD, PBE+MBD-NL, HSE+MBD, and HSE+MBD-NL. The interlayer distances, *d*, used in the calculations are not necessarily the optimized equilibrium values.

Stacking	Method	*d* (Å)	BE (meV/atom)	Reference
AB	DMC	3.384	17.7(9)	22
AA	DMC	3.495	11.5(9)	22
AB	PBE+MBD (old)	3.37	22.8	23
AB	PBE+MBD (old)	3.35	22.4	This work
AB	HSE+MBD (old)	3.35	24.7	This work
AB	PBE+MBD-NL	3.35	17.8	This work
AB	HSE+MBD-NL	3.36	20.39	This work
AA	HSE+MBD-NL	3.62	15.68	This work

A similar picture arises for the case of bilayer *h*-BN (see **Table S3**), where the MBD-NL results are in better agreement (within 2.6 meV/atom) with the DMC value than the corresponding MBD results (within 6.4 meV/atom). Specifically, the HSE+MBD-NL results are in remarkable agreement (within 0.6 meV/atom) with the DMC value.

Table S3. Binding energy (BE) of bilayer *h*-BN obtained using DMC, PBE+MBD, PBE+MBD-NL, HSE+MBD, and HSE+MBD-NL. The interlayer distances, *d*, used in the calculations are not necessarily the optimized equilibrium values.

Stacking	Method	*d* (Å)	BE (meV/atom)	Reference
AB	DMC	~3.375	20.4	24
AB	PBE+MBD (old)	3.37	24.7	23
AB	PBE+MBD (old)	3.4	24.8	This work
AB	HSE+MBD (old)	3.4	26.8	This work
AB	PBE+MBD-NL	3.4	17.8	This work
AB	HSE+MBD-NL	3.4	19.8	This work

Turning to consider bulk systems, **Table S4** compares experimental binding energies of graphite,

with values obtained using QMC, the RPA, and DFT results calculated at the PBE+MBD, PBE+MBD-NL, HSE+MBD, and HSE+MBD-NL level of theory. The QMC (56±5 meV/atom) and RPA (48 meV/atom) results are within the higher range of experimentally available values (31-54 meV/atom). The PBE+MBD values compare well with the RPA results (within <1 meV/atom), whereas the HSE+MBD value is in better agreement with the QMC result (within 2.5 meV/atom). Adding the NL correction reduces the calculated MBD binding energies by ~8 meV/atom making the HSE+MBD-NL results in good agreement with the RPA value, to within 2.6 meV/atom. Notably, all the MBD and MBD-NL results considered are well within the experimentally available range of binding energies.

Table S4. Binding energy (BE) of bulk graphite obtained experimentally and computationally using QMC, RPA, PBE+MBD, PBE+MBD-NL, HSE+MBD, and HSE+MBD-NL. The interlayer distances, *d*, used in the calculations are not necessarily the optimized equilibrium values.

Stacking	Method	*d* (Å)	BE (meV/atom)	Reference
AB	QMC	3.426	56±5	25
AB	RPA	3.34	48	26
AB	PBE+MBD (old)	3.34	48.2	23
AB	PBE+MBD (old)	3.35	48.8	This work
AB	HSE+MBD (old)	3.35	53.5	This work
AB	PBE+MBD-NL	3.35	40.1	This work
AB	PBE+MBD-NL	N/A	40.6	13
AB	HSE+MBD-NL	3.35	45.4	This work
	Experiments		52 ± 5	27
	Experiments		43 ± 5	28
	Experiments		35^{+15}_{-10}	29
	Experiments		31 ± 2	30
	Experiments		64±3	31
	Experiments		37.1±0.8	32

Finally, **Table S5** compares the binding energy of bulk *h*-BN calculated using the RPA to DFT values

obtained at the PBE+MBD and PBE+MBD-NL level of theory. While the MBD results (both with PBE and HSE) are considerably higher (by 13-17.5 meV/atom) than the RPA value of 39.3 meV/atom, the MBD-NL results are in good agreement with the RPA value (within 0.2 and 3.8 meV/atom for the PBE+MBD-NL and HSE+MBD-NL values, respectively). Here, as well, it should be noted that part of the differences may be attributed to the different interlayer distances used for the calculation.

Table S5. Binding energy (BE) of bulk *h*-BN obtained using RPA, PBE+MBD, PBE+MBD-NL, HSE+MBD, and HSE+MBD-NL. The interlayer distances, *d*, used in the calculations are not necessarily the optimized equilibrium values.

Stacking	Method	*d* (Å)	BE (meV/atom)	Reference
AB	RPA	3.34	39.3	33
AB	PBE+MBD (old)	3.33	52.4	23
AB	PBE+MBD (old)	3.4	52.9	This work
AB	HSE+MBD (old)	3.4	56.7	This work
AB	PBE+MBD-NL	3.4	39.1	This work
AB	PBE+MBD-NL	N/A	39.1	13
AB	HSE+MBD-NL	3.4	43.1	This work
AA'	HSE+MBD-NL	3.24	44.8	This work

Altogether, we find that the PBE+MBD-NL and HSE+MBD-NL DFT approaches show overall good agreement with available experimental and high-accuracy calculation results for the binding energies of bilayer and bulk graphene and *h*-BN. This signifies the reliability of these approaches for obtaining reference binding energy datasets for layered materials.

1.2. Sliding energy surfaces

The left columns of **Figure S3** and **Figure S4** present the sliding potential energy surfaces (PES) calculated at fixed interlayer distances using HSE+MBD-NL for bulk and bilayer interfaces, respectively. The corresponding ILP data appear in the middle column of both figures and the differences between the reference DFT data and the ILP results are presented in the right columns.

For all three systems, HSE+MBD-NL predicts somewhat lower PES corrugation than HSE+MBD. The ILP fitting is in good qualitative and quantitative agreement with the DFT reference data. Specifically, the maximal deviation between the DFT reference and the ILP results for bulk graphite and bilayer graphene is 4.3% and 5.5% of the overall PES corrugation, respectively. The corresponding differences for bulk (bilayer) *h*-BN and the heterogeneous structures are 3.3 % (1.4 %) and 1.3 % (2.1 %), respectively.

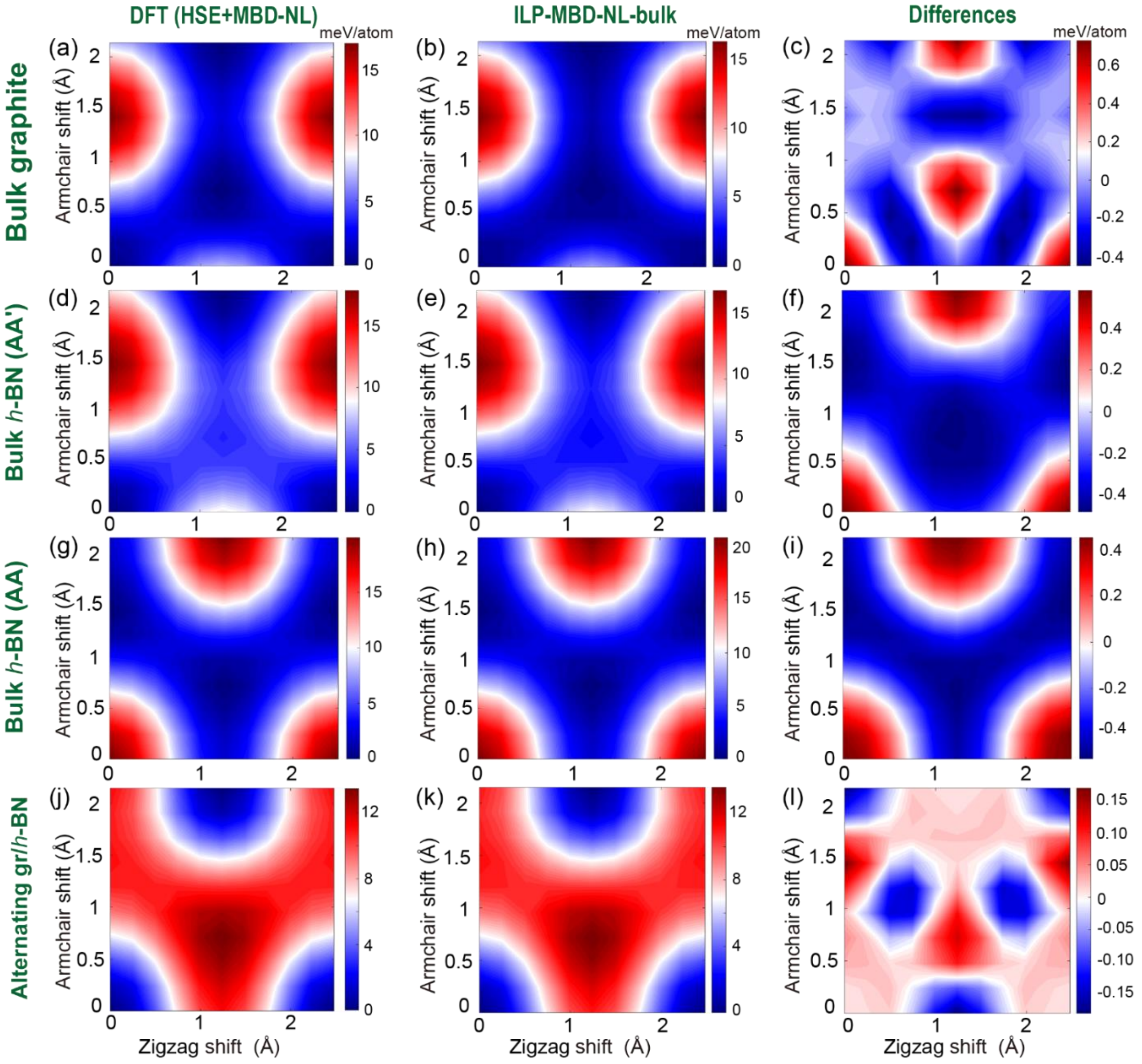

Figure S3. *Sliding energy surfaces of the periodic bulk structures considered, calculated at an interlayer distance of 3.3 Å. The first and second columns present the sliding energy surfaces of bulk graphite (a, b), bulk h-BN with anti-parallel (AA'; d, e) and parallel (AA; g, h) configurations, and alternating C-stacked graphene/h-BN (j, k) systems,[9] calculated using HSE+MBD-NL and the corresponding ILP parameterization, respectively. The third column presents their differences. The parameters of* ***Table S6*** *are used for the ILP calculations. The reported energies are measured relative to value obtained for the infinitely separated layers and are normalized by the total number of atoms in the unit-cell (4 atoms).*

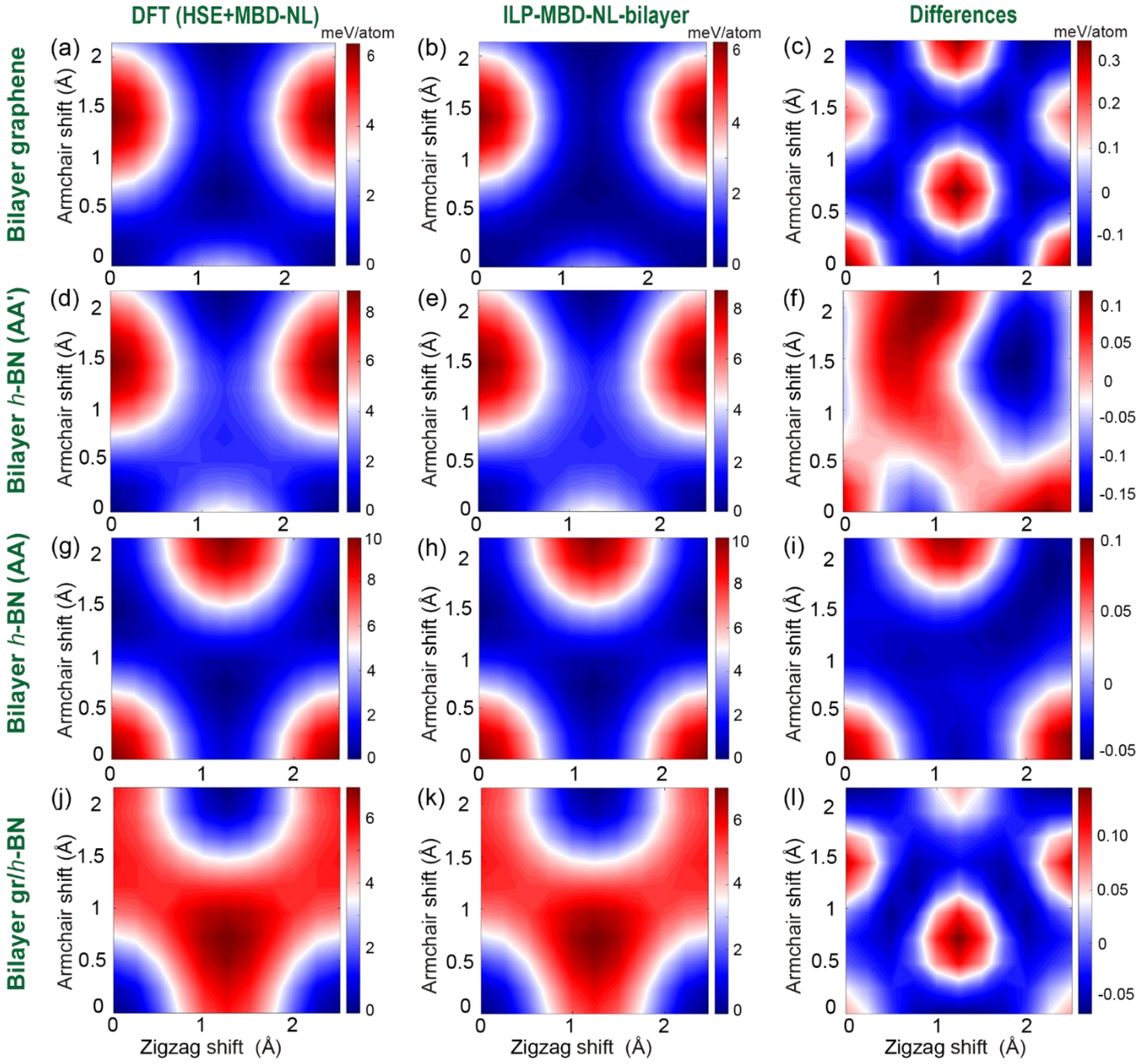

Figure S4. *Sliding energy surfaces of the laterally periodic bilayer structures considered. The first and second columns present the sliding energy surface of bilayer graphene (a, b) with an interlayer distance of 3.4 Å, bilayer h-BN with anti-parallel (AA'; d, e) and parallel (AA; g, h) configurations, and bilayer C-stacked graphene/h-BN (j, k) systems*[9] *both with an interlayer distance of 3.3 Å, calculated using HSE+MBD-NL and the corresponding ILP parameterization, respectively. The third column presents their differences. The parameters of* ***Table S7*** *are used for the ILP calculations. The reported energies are measured relative to value obtained for the infinitely separated layers and are normalized by the total number of atoms in the unit-cell (4 atoms).*

The fitted MBD-NL graphene and h-BN ILP parameters appear in **Table S6** (bulk) and **Table S7** (bilayer) below.

Table S6. *List of ILP parameter values for bulk graphite and bulk h-BN systems that are periodic in both lateral and vertical directions. The training set includes all HSE+MBD-NL bulk binding energy curves and sliding energy surfaces appearing in* ***Figure S2-Figure S4****. A value of* $R_{cut} = 16$ Å *is used throughout. Note that all parameters are symmetric with respect to indices interchange, e.g.* $\alpha_{B,N} = \alpha_{N,B}$.

	β_{ij} (Å)	α_{ij}	γ_{ij} (Å)	ε_{ij} (meV)	C_{ij} (meV)	d_{ij}	$s_{R,ij}$	$r_{eff,ij}$ (Å)	$C_{6,ij}$ (eV•Å^6)	λ_{ij} (Å^{-1})
**C-C**	3.0875	7.1844	1.2573	2.101e-3	46.4404	19.9987	0.8642	3.4275	23.4328	--
**B-B**	3.2810	4.3117	1.8734	15.2062	23.1795	16.5196	0.8654	3.3260	49.4984	0.70
**N-N**	3.6079	10.2279	1.6959	0.0253	16.6118	24.6309	1.0166	3.1103	14.8107	0.69
**B-N**	3.0910	3.9352	2.9525	0.0069	0.0023	12.0044	0.9542	3.4077	24.6706	0.694982
**C-B**	4.0402	4.7202	1.2831	2.8494	3.0700	39.8423	0.7859	3.4330	39.2634	--
**C-N**	3.2332	8.1656	1.4147	20.1928	25.2544	27.9332	0.9462	3.4844	19.9631	--

Table S7. *List of ILP parameter values for bilayer graphene and bilayer h-BN systems that are periodic in the lateral directions. The training set includes all HSE+MBD-NL bilayer binding energy curves and sliding potential surfaces appearing in* ***Figure S2-Figure S4****. A value of* $R_{cut} = 16$ Å *is used throughout. Note that all parameters are symmetric with respect to indices interchange, e.g.* $\alpha_{B,N} = \alpha_{N,B}$

	β_{ij} (Å)	α_{ij}	γ_{ij} (Å)	ε_{ij} (meV)	C_{ij} (meV)	d_{ij}	$s_{R,ij}$	$r_{eff,ij}$ (Å)	$C_{6,ij}$ (eV•Å^6)	λ_{ij} (Å^{-1})
**C-C**	3.2235	8.2312	1.1795	9.652e-5	31.3750	19.9997	0.8307	3.5144	20.1470	--
**B-B**	3.1573	9.5005	1.6242	18.2735	5.3371	13.1189	0.7806	3.6929	42.1291	0.70
**N-N**	3.1230	8.4935	1.7099	0.5192	68.7316	12.1406	0.7249	3.3617	16.7300	0.69
**B-N**	3.6646	4.6045	3.7885	7.9142	0.0138	14.4745	0.7713	3.6102	26.3673	0.694982
**C-B**	3.1188	10.5118	1.4452	0.0059	7.1244	15.6605	0.8340	3.5667	22.9953	--
**C-N**	3.0451	7.5235	1.4804	0.0094	57.8603	19.9894	0.8066	3.5843	14.8577	--

1.3. Compressibility of graphite and hexagonal boron nitride

The simulation results obtained using the HSE+MBD and HSE+MBD-NL parameterized ILP for graphite and bulk *h*-BN under hydrostatic pressure are presented in **Figure S5**, along with the experimental *c*-*P* curves. We find that the HSE+MBD-NL parametrized ILP *c*-*P* curves compare well with experimental measurements,[34-40] for both bilayer and bulk parameterizations, over the entire range of pressures investigated. Notably, despite the differences discussed above in the binding energies obtained by MBD and MBD+NL, all considered ILP parametrizations predict very similar *c*-*P* curves even in the deep sub-equilibrium regime, where bulk graphite and *h*-BN are compressed down to 0.6 of their equilibrium interlayer distance.

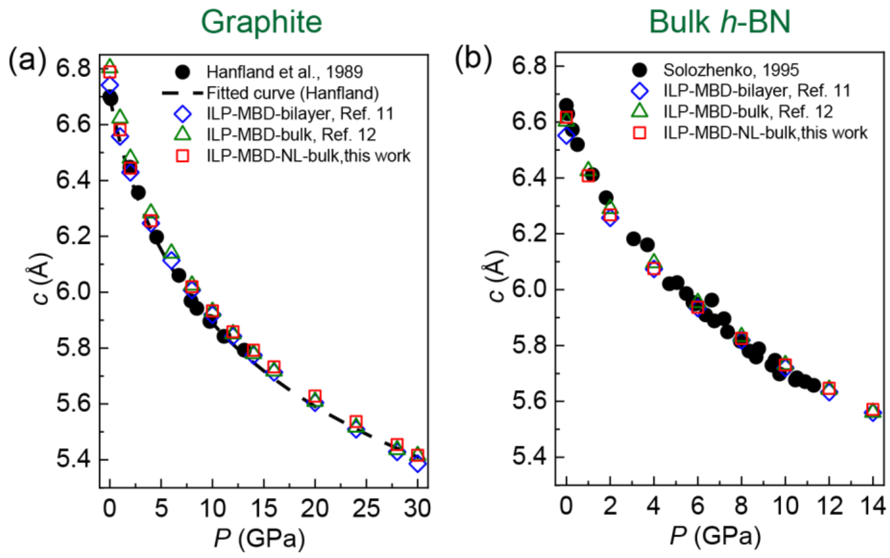

Figure S5. *Measured and computed pressure dependence of the c lattice parameter of (a) bulk graphite and (b) bulk h-BN. Full symbols represent experimental results*[35, 37] *and open points represent NPT simulation results for different parameterizations of the ILP, as specified in the corresponding set labels. Error bars for the simulated data, obtained from the temporal standard deviation of the interlayer distance thermal fluctuations at equilibrium, are smaller than the symbol width.*

The bulk modulus extracted from the pressure-volume (*P*-*V*) curves (see **Figure S6**) using Eq. 8 of the main text is compared to available experimental data in **Table S8**. Here, apart from the Murnaghan equation, two other equations of state (EOS) are also used to fit the *P*-*V* curve: (i) The Birch-Murnaghan equation (eq S1)[41, 42] and (ii) The Vinet equation (eq S2),[43, 44] which take the following forms:

$$P = 3B_V^0\xi(1+2\xi)^{5/2}\left[1-\frac{3}{2}(4-B_V')\xi\right],\ \ \xi=\frac{1}{2}\left[\left(\frac{V}{V_0}\right)^{-\frac{2}{3}}-1\right]. \tag{S1}$$

$$P = 3B_V^0\frac{(1-X)}{X^2}\exp\left[\frac{3}{2}(B_V'-1)(1-X)\right],\ \ X=\left(\frac{V}{V_0}\right)^{\frac{1}{3}}. \tag{S2}$$

As in the Murnaghan equation, these two EOS also assume that B_V varies with pressure (hence the inclusion of B_V'). Nonetheless, they differ in their description of the dependence of B_V on the pressure, by assuming that it is linear, polynomial, and exponential for the Murnaghan, Birch–Murnaghan, and Vinet EOS, respectively.

Comparing the values of the bulk modulus predicted for graphite by the equilibrium molecular dynamics simulations,[12] we found that the MBD-NL ILP parametrizations predict somewhat lower values compared to the original MBD ILP parametrizations but still in good agreement with the experimental values. Considering the bulk modulus derivative with respect to the external pressure, we find that all MBD force-field parameterizations provide good agreement with the lower experimental value of 8.9±0.1. The only outlier within our test-set is the TS parameterized ILP, which underestimates the bulk modulus pressure derivative by nearly 30%.

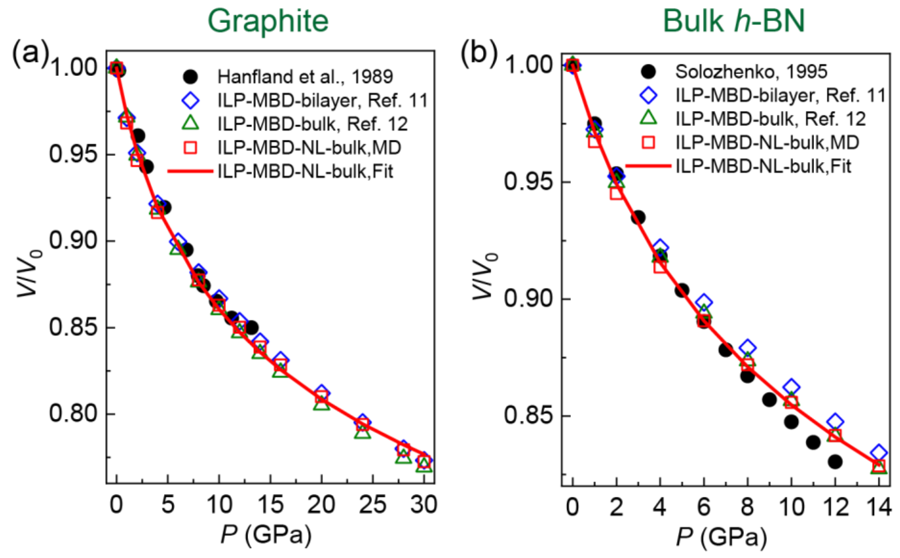

Figure S6. *Pressure dependence of the normalized volume V/V_0 of bulk graphite and bulk h-BN. The open points are the NPT simulations results for different parameterizations of the ILP potentials. The solid lines are fitted curves generated using Eq. 8 of the main text. Full black circles represent available experimental data.*[35, 37]

The experimental values of the bulk modulus and its pressure derivative for bulk *h*-BN are more scattered than those for graphite, ranging from 17.6 GPa to 36.7 GPa and 5.6 to 19.5, respectively. Therefore, it is difficult to draw a definite conclusion regarding the method that provides best results.

Nevertheless, all methods listed in **Table S8**, yield values within the experimentally measured range. Overall, the ILP parameterization based on both HSE+MBD-NL and HSE+MBD methods are able to capture the bulk properties of graphite and bulk *h*-BN well.

Table S8. *Bulk moduli obtained by fitting equilibrium molecular dynamics simulation data with different equations of state (see Eqs. S1-S2 and Eq. 8 of the main text) for graphite and bulk h-BN. Experimental values are presented for comparison.*

Material	Methods	Murnaghan		Birch-Murnaghan		Vinet	
		B_V^0 (GPa)	B_V'	B_V^0 (GPa)	B_V'	B_V^0 (GPa)	B_V'
Graphite	Experiments	33±2[a]	12.3±0.7[a]	--	--	--	--
		33.8±0.3[b]	8.9±0.1[b]	--	--	--	--
		--	--	--	--	30.8±2[c]	--
	ILP-MBD-NL-bulk	32±3	8.8±0.8	27±2	15±1	32±2	10.4±0.6
	ILP-MBD-NL-bilayer	29±2	8.9±0.5	21.7±0.4	18.3±0.4	27.7±0.9	11.1±0.3
	ILP-MBD-bulk[12]	34±1	8.1±0.3	27±1	14.2±0.7	31.5±0.8	10.2±0.2
	ILP-TS-bulk[12]	55±2	6.2±0.3	53±0.9	7.5±0.2	53.4±0.9	7.4±0.2
	ILP-MBD-bilayer-refined[11]	36±3	8.1±0.6	33±2	12.2±0.9	36±2	9.6±0.5
	ILP-MBD-bilayer-original[10]	33±1	8.5±0.3	25.5±0.8	16.3±0.7	30.7±0.3	10.8±0.1
	KC-MBD-bilayer-refined[11]	35±2	7.7±0.3	30.5±0.5	12.0±0.3	33.5±0.7	9.5±0.2
	KC-original[45]	37±2	8.9±0.4	29.3±0.6	16.7±0.4	35.1±0.7	11.1±0.2
Bulk *h*-BN	Experiments	22±4[a]	18±3[a]	--	--	--	--
		36.7±0.5[d]	5.6±0.2[d]	--	--	--	--
		--	--	17.6±0.8[e]	19.5±3.4[e]	--	--
		--	--	27.6±0.5[f]	10.5±0.5[f]	--	--
	ILP-MBD-NL-bulk	28±2	9.5±0.9	25±2	15±1	28±2	10.9±0.8
	ILP-MBD-NL-bilayer	31±2	8.6±0.7	29±1	12.0±0.7	31±1	10.0±0.5
	ILP-MBD-bulk[12]	33±2	7.8±0.6	31±1	10.2±0.8	32±1	9.0±0.5
	ILP-TS-bulk[12]	35±2	8.7±0.6	33±1	12.0±0.7	34±1	10.0±0.5
	ILP-MBD-bilayer-refined[11]	35±2	8.0±0.6	33±1	10.5±0.7	34±1	9.2±0.5
	ILP-MBD-bilayer-original[10]	38±3	8.7±0.9	36±2	11±1	38±2	9.7±0.9

[a]ref 34, [b]ref 35, [c]ref 36, [d]ref 37, [e]ref 38, [f]ref 46

1.4. Phonon spectra for graphite and bulk h-BN

Finally, we present in **Figure S7** the phonon dispersion curves of graphite and bulk *h*-BN calculated using the HSE+MBD-NL parametrized ILP at zero pressure and temperature and compared them with the experimental data reported in Refs. [47] and [48]. We find that the dispersion of the low energy out-of-plane (ZA) branches, which are related to the soft flexural modes of the layers, is well described by both HSE+MBD-NL and HSE+MBD parametrized ILP, for graphite and for bulk *h*-BN. Notably, despite the abovementioned differences in the predicted binding energies, the phonon spectra obtained by the two DFT parametrizations are comparable. The larger deviations from the experimental data observed for the high energy transverse (TO) and longitudinal (LO) optical modes, are attributed mainly to the intralayer potential used in our simulations. More details can be found in Refs. [49, 50], as well as in Sec. 4.

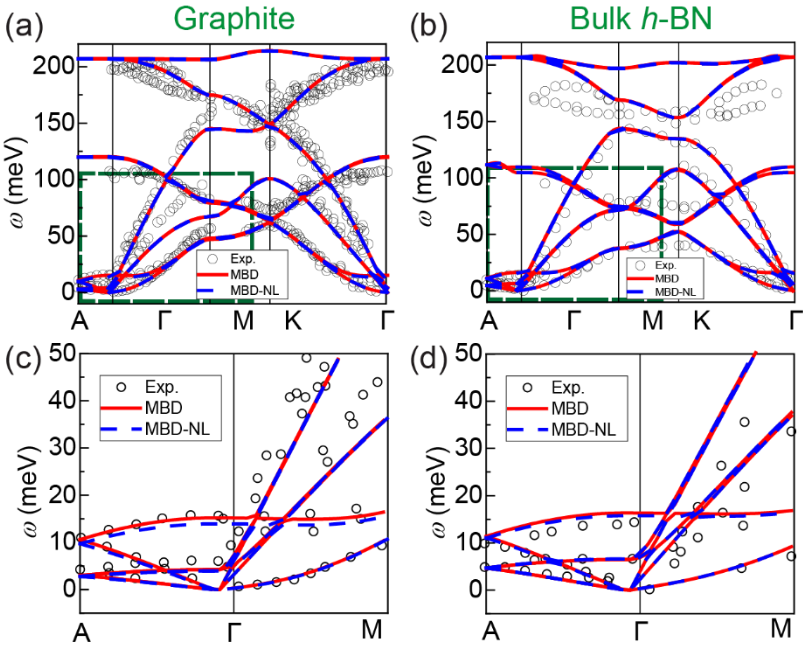

Figure S7. *Phonon dispersion of (a) bulk graphite and (b) bulk h-BN. Red solid lines and blue dashed lines are dispersion curves calculated using the ILP parameterized against HSE+MBD*[12] *and HSE+MBD-NL (***Table S6***) bulk reference data, respectively. Experimental results of bulk graphite*[47] *and bulk h-BN*[48] *are given by open black circles. Panels (c) and (d) show a zoom-in on the low energy phonon modes around the Γ-point (green rectangles in panels (a) and (b)) for graphite and h-BN, respectively.*

2. Convergence Tests of the Reference DFT Calculations

To confirm convergence of the DFT results, we performed a series of BE calculations with different *k*-grid densities for both bilayer and bulk configurations, and vacuum sizes for the bilayer system, keeping all other settings identical to those used to obtain the results appearing in the main text. The results are presented in **Figure S8** and **Figure S9** for MoS_2 and graphene and *h*-BN, respectively, where the parameter values used to obtain the results appearing in the main text are marked in red. For MoS_2, a BE convergence of ~0.05 meV/atom with respect to the two parameters is found for both bilayer and bulk configurations. For bilayer graphene and bilayer graphene/*h*-BN the BE convergence is ~ 0.4 meV/atom, whereas for bilayer *h*-BN, bulk graphite, bulk h-BN, and alternating bulk graphene/*h*-BN the BE convergence is ~0.05 meV/atom. We further evaluated the convergence of the calculated BEs with respect to the choice of basis-set. Taking bilayer AA' stacked MoS_2 at an interlayer distance (distance between two Mo atomic planes, see **Figure 1** in the main text) of 6.2 Å as an example, the BE obtained using the tier-2 basis-set (21.0 meV/atom) is only 1.4% larger than that obtained using the tier-3 basis-set (20.7 meV/atom). This suggests the tier-2 basis-set used in our calculations provides sufficiently converged BE results.

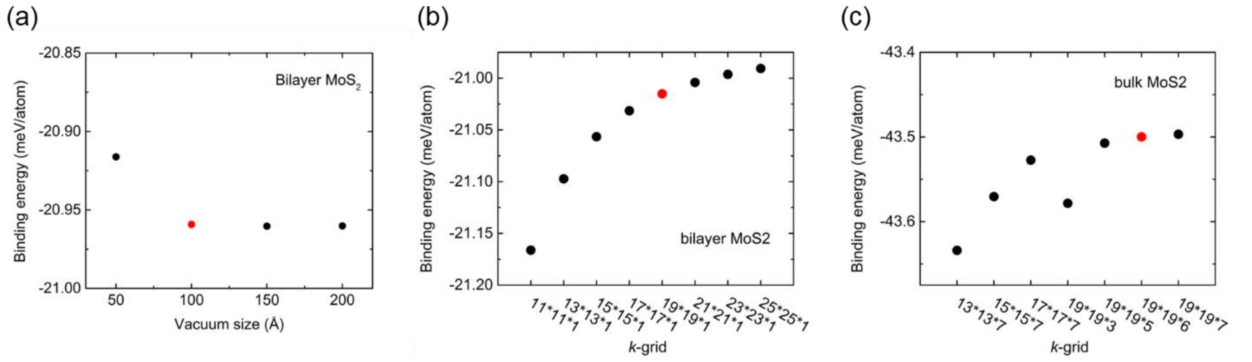

Figure S8. *AA' stacked MoS_2 BE convergence tests with respect to (a) the bilayer unit-cell vacuum size, (b) bilayer k-grid density, and (c) bulk k-grid density. The red colored symbols mark the values used to obtain the results presented in the main text. The k-grid in the calculations in (a) was chosen as 15×15×1 and the interlayer distance was fixed at 6.21 Å. The interlayer distance in the calculations in (b) and (c) was fixed at 6.20 Å.*

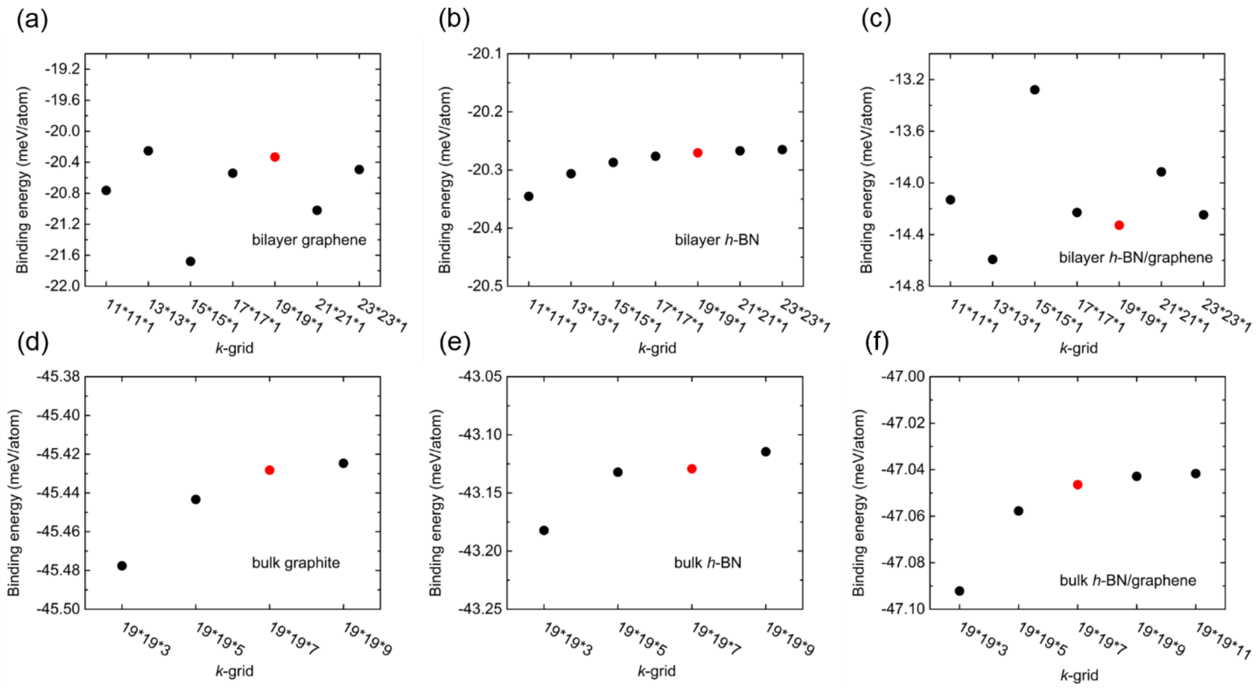

Figure S9. *BE convergence tests for rigid bilayer (upped panels) and bulk (lower panels) graphene (a),(d), h-BN (b),(e), and their heterostructures (c),(f) with respect to k-grid density. The red colored symbols represent the values used to obtain the results appearing in the main text. In panel (a) the interlayer distance of the AB stacked bilayer graphene is taken as 3.4 Å. In panel (b) the interlayer distance of the AA' stacked bilayer h-BN is taken as 3.3 Å. In panel (c) the interlayer distance of the A stacked graphene/h-BN bilayer is taken as 3.3 Å. The interlayer distances used for the AB staked graphite [panel (d)], AA' stacked bulk h-BN [panel (e)], and C stacked alternating graphene/h-BN bulk [panel (e)] calculations are 3.35 Å, 3.4 Å, and 3.3 Å, respectively. See* ***Figure S1*** *for an illustration of various high-symmetry stacking modes of graphene and h-BN interfaces.*

3. Interlayer Potential Parameters for bulk MoS_2

In this work, all reference data were obtained using dispersion-augmented density functional theory (DFT) calculations, which are based on the screened-exchange hybrid functional of Heyd, Scuseria, and Ernzerhof (HSE).[1-4] In previous studies, we employed both many-body dispersion (MBD)[5, 6] and Tkatchenko-Scheffler (TS) corrections[7, 8] along with the HSE functional. The former scheme (HSE + MBD) was shown to provide a good balance between accuracy and computational burden for calculating binding energy (BE) curves and sliding potential energy surfaces (PES) for bilayer graphene, h-BN, and their heterojunctions.[9, 10] In our recent studies, we extended the interlayer potential (ILP) parameterization to accurately treat also the sub-equilibrium regime.[11, 12] In the present study, to evaluate the properties of transition-metal dichalcogenides (TMDs) materials, we performed reference DFT calculations adopting the newly developed nonlocal many-body dispersion method (MBD-NL),[13] which is applicable for highly polarizable materials. The ILP parameters obtained using the fitting procedure described in Refs. 11, 12 against the bulk MoS_2 DFT reference data are presented in **Table S9**. The resulting BE curves and PESs appear in **Figures 2-4** of the main text.

Table S9. *List of ILP parameter values for bulk* MoS_2*, which is periodic in both lateral and vertical directions. The training set includes all HSE + MBD-NL bulk binding energy curves and sliding potential surfaces appearing in* ***Figures 2-4*** *of the main text. A value of* $R_{\mathrm{cut}} = 16\,\text{Å}$ *is used throughout. Note that all parameters are symmetric with respect to indices interchange, e.g.* $\alpha_{Mo,S} = \alpha_{S,Mo}$.

	β_{ij} (Å)	α_{ij}	γ_{ij} (Å)	ε_{ij} (meV)	C_{ij} (meV)	d_{ij}	$s_{R,ij}$	$r_{eff,ij}$ (Å)	$C_{6,ij}$ (eV•Å^6)
**Mo-Mo**	5.5795	9.3777	2.0272	144.1518	97.9786	89.4376	2.0590	5.1221	491.8503
**S-S**	3.1614	8.0933	1.9531	4.5868	118.0655	58.8094	0.2154	4.2996	148.8112
**Mo-S**	3.6272	19.9714	7.5850	76.1019	3.3175	45.7203	0.9475	4.4104	150.5979

4. Effect of Intra- and Inter-Layer Potentials on the Calculated Phonon Spectra of MoS_2

In this section, we illustrate the effect of intralayer potential on the calculated phonon dispersion curves of bulk MoS_2 at zero pressure and temperature.[14] To this end, we use two types of intra-layer MoS_2 force-fields: (i) the second-generation REBO potential,[15, 16] and (ii) the Stillinger-Weber (SW) potential.[17]. **Figure S10**a-b show the comparison between the phonon dispersion curves of bulk and single layer MoS_2 calculated using both intralayer force fields. The comparison demonstrates that the intralayer potential mainly influences the high-energy phonon dispersion curves, whereas the low energy phonons are weakly affected by the choice of intralayer potential (see **Figure S10**c-d).

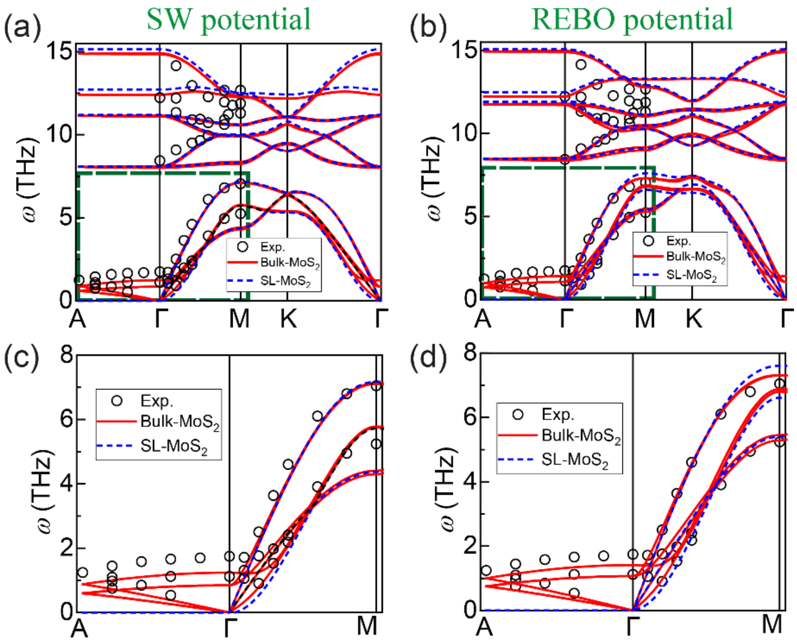

Figure S10. *Phonon dispersion of MoS_2 calculated using the (a) Stillinger-Weber (SW) potential,[17] and (b) REBO potential.[15, 16] Red solid lines and blue dashed lines are dispersion curves calculated for bulk (using the ILP) and single layer MoS_2, respectively. Experimental results for bulk MoS_2 are given by open black circles.[14] Panels (c) and (d) provide a zoom-in on the low energy phonon modes around the Γ-point (green rectangles in panels (a) and (b)) for the SW and the REBO potentials, respectively.*

Next, to evaluate the effect of choice of interlayer potential we compared the phonon dispersion of bulk MoS_2 calculated using the ILP with that calculated using the isotropic Lennard-Jones (LJ) potential.[15] The results for both the Stillinger-Weber (SW)[17] and the REBO[15, 16] intralayer potentials are presented in **Figure S11**a, and **Figure S11**b, respectively.

As expected, the choice of interlayer potential affects mostly the low energy phonon dispersion that corresponds to interlayer phonon modes. Zooming in on this region (see **Figure S11**c-d) reveals that the ILP dispersion curves agree better with the experimental data for bulk MoS_2[14] than the LJ predictions. This further signifies the importance of using a dedicated anisotropic potential to describe the interlayer interactions in TMDs.

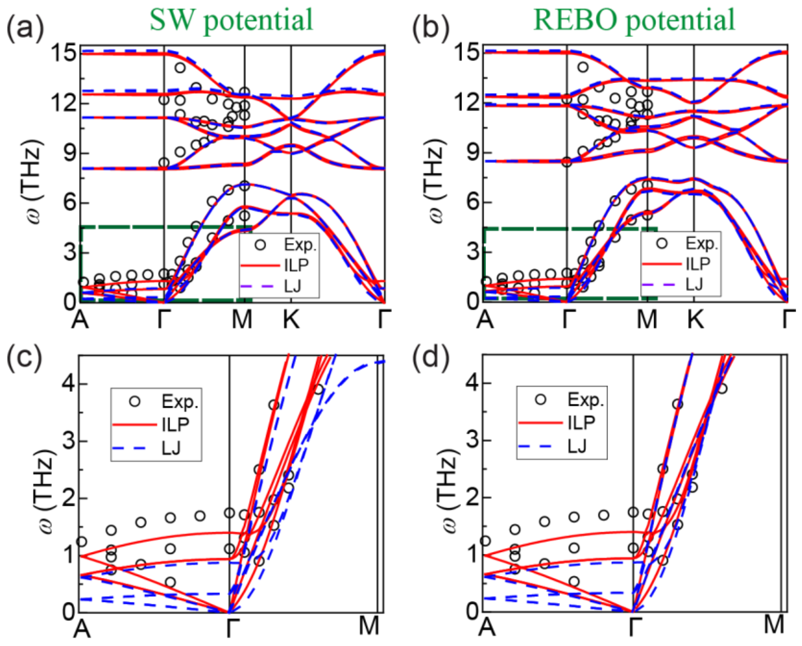

Figure S11. *Phonon dispersion of bulk MoS_2 calculated using the ILP (red lines) and the LJ potential (dashed blue lines) with the (a) Stillinger-Weber (SW) potential,[17] and (b) REBO potential.[15, 16] Experimental results for bulk MoS_2 are given by open black circles.[14] Panels (c) and (d) provide a zoom-in on the low energy phonon modes around the Γ-point (green rectangles in panels (a) and (b)) for the SW and the REBO intralayer potentials, respectively.*

References

(1) Heyd, J.; Scuseria, G. E.; Ernzerhof, M. Hybrid functionals based on a screened Coulomb potential. *J. Chem. Phys.* **2003**, *118*, 8207-8215.

(2) Heyd, J.; Scuseria, G. E. Assessment and validation of a screened Coulomb hybrid density functional. *J. Chem. Phys.* **2004**, *120*, 7274-7280.

(3) Heyd, J.; Scuseria, G. E. Efficient hybrid density functional calculations in solids: Assessment of the Heyd–Scuseria–Ernzerhof screened Coulomb hybrid functional. *J. Chem. Phys.* **2004**, *121*, 1187-1192.

(4) Heyd, J.; Scuseria, G. E.; Ernzerhof, M. Erratum: "Hybrid functionals based on a screened Coulomb potential" [J. Chem. Phys. 118, 8207 (2003)]. *J. Chem. Phys.* **2006**, *124*, 219906.

(5) Tkatchenko, A.; DiStasio, R. A.; Car, R.; Scheffler, M. Accurate and Efficient Method for Many-Body van der Waals Interactions. *Phys. Rev. Lett.* **2012**, *108*, 236402.

(6) Ambrosetti, A.; Reilly, A. M.; DiStasio Jr., R. A.; Tkatchenko, A. Long-range correlation energy calculated from coupled atomic response functions. *J. Chem. Phys.* **2014**, *140*, 18A508.

(7) Tkatchenko, A.; Scheffler, M. Accurate Molecular Van Der Waals Interactions from Ground-State Electron Density and Free-Atom Reference Data. *Phys. Rev. Lett.* **2009**, *102*, 073005.

(8) Marom, N.; Tkatchenko, A.; Scheffler, M.; Kronik, L. Describing Both Dispersion Interactions and Electronic Structure Using Density Functional Theory: The Case of Metal−Phthalocyanine Dimers. *J. Chem. Theory Comput.* **2010**, *6*, 81-90.

(9) Leven, I.; Maaravi, T.; Azuri, I.; Kronik, L.; Hod, O. Interlayer Potential for Graphene/h-BN Heterostructures. *J. Chem. Theory Comput.* **2016**, *12*, 2896-905.

(10) Maaravi, T.; Leven, I.; Azuri, I.; Kronik, L.; Hod, O. Interlayer Potential for Homogeneous Graphene and Hexagonal Boron Nitride Systems: Reparametrization for Many-Body Dispersion Effects. *J. Phys. Chem. C* **2017**, *121*, 22826-22835.

(11) Ouyang, W.; Mandelli, D.; Urbakh, M.; Hod, O. Nanoserpents: Graphene Nanoribbon Motion on Two-Dimensional Hexagonal Materials. *Nano Lett.* **2018**, *18*, 6009-6016.

(12) Ouyang, W.; Azuri, I.; Mandelli, D.; Tkatchenko, A.; Kronik, L.; Urbakh, M.; Hod, O. Mechanical and Tribological Properties of Layered Materials under High Pressure: Assessing the Importance of Many-Body Dispersion Effects. *J. Chem. Theory Comput.* **2020**, *16*, 666-676.

(13) Hermann, J.; Tkatchenko, A. Density Functional Model for van der Waals Interactions: Unifying Many-Body Atomic Approaches with Nonlocal Functionals. *Phys. Rev. Lett.* **2020**, *124*, 146401.

(14) Wakabayashi, N.; Smith, H. G.; Nicklow, R. M. Lattice dynamics of hexagonal MoS_2 studied by neutron scattering. *Phys. Rev. B* **1975**, *12*, 659-663.

(15) Liang, T.; Phillpot, S. R.; Sinnott, S. B. Parametrization of a reactive many-body potential for Mo-S systems. *Phys. Rev. B* **2009**, *79*, 245110.

(16) Liang, T.; Phillpot, S. R.; Sinnott, S. B. Erratum: Parametrization of a reactive many-body potential for Mo--S systems [Phys. Rev. B 79, 245110 (2009)]. *Phys. Rev. B* **2012**, *85*, 199903.

(17) Jin-Wu, J. Parametrization of Stillinger–Weber potential based on valence force field model: application to single-layer MoS_2 and black phosphorus. *Nanotechnology* **2015**, *26*, 315706.

(18) Blum, V.; Gehrke, R.; Hanke, F.; Havu, P.; Havu, V.; Ren, X.; Reuter, K.; Scheffler, M. Ab initio molecular simulations with numeric atom-centered orbitals. *Comput. Phys. Commun.* **2009**, *180*, 2175-2196.

(19) Havu, V.; Blum, V.; Havu, P.; Scheffler, M. Efficient O(N) integration for all-electron electronic structure calculation using numeric basis functions. *J. Comput. Phys.* **2009**, *228*, 8367-8379.

(20) Hariharan, P. C.; Pople, J. A. The influence of polarization functions on molecular orbital hydrogenation energies. *Theoretica chimica acta* **1973**, *28*, 213-222.

(21) Frisch, M.; Trucks, G.; Schlegel, H.; Scuseria, G.; Robb, M.; Cheeseman, J.; Scalmani, G.; Barone, V.; Mennucci, B.; Petersson, G. Gaussian 09, revision A. 02; Gaussian, Inc: Wallingford, CT, 2009.

(22) Mostaani, E.; Drummond, N. D.; Fal'ko, V. I. Quantum Monte Carlo calculation of the binding energy of bilayer graphene. *Phys. Rev. Lett.* **2015**, *115*, 115501.

(23) Gao, W.; Tkatchenko, A. Sliding Mechanisms in Multilayered Hexagonal Boron Nitride and Graphene: The Effects of Directionality, Thickness, and Sliding Constraints. *Phys. Rev. Lett.* **2015**, *114*, 096101.

(24) Hsing, C.-R.; Cheng, C.; Chou, J.-P.; Chang, C.-M.; Wei, C.-M. Van der Waals interaction in a boron nitride bilayer. *New J. Phys.* **2014**, *16*, 113015.

(25) Spanu, L.; Sorella, S.; Galli, G. Nature and strength of interlayer binding in graphite. *Phys. Rev. Lett.* **2009**, *103*, 196401.

(26) Lebègue, S.; Harl, J.; Gould, T.; Ángyán, J. G.; Kresse, G.; Dobson, J. F. Cohesive Properties and Asymptotics of the Dispersion Interaction in Graphite by the Random Phase Approximation. *Phys. Rev. Lett.* **2010**, *105*, 196401.

(27) Zacharia, R.; Ulbricht, H.; Hertel, T. Interlayer cohesive energy of graphite from thermal desorption of polyaromatic hydrocarbons. *Phys. Rev. B* **2004**, *69*, 155406.

(28) Girifalco, L. A.; Lad, R. A. Energy of Cohesion, Compressibility, and the Potential Energy Functions of the Graphite System. *J. Chem. Phys.* **1956**, *25*, 693-697.

(29) Benedict, L. X.; Chopra, N. G.; Cohen, M. L.; Zettl, A.; Louie, S. G.; Crespi, V. H. Microscopic determination of the interlayer binding energy in graphite. *Chem. Phys. Lett.* **1998**, *286*, 490-496.

(30) Liu, Z.; Liu, J. Z.; Cheng, Y.; Li, Z.; Wang, L.; Zheng, Q. Interlayer binding energy of graphite: A mesoscopic determination from deformation. *Phys. Rev. B* **2012**, *85*, 205418.

(31) Wang, W.; Dai, S.; Li, X.; Yang, J.; Srolovitz, D. J.; Zheng, Q. Measurement of the cleavage energy of graphite. *Nat. commun.* **2015**, *6*, 7853.

(32) Koren, E.; Lörtscher, E.; Rawlings, C.; Knoll, A. W.; Duerig, U. Adhesion and friction in mesoscopic graphite contacts. *Science* **2015**, *348*, 679-683.

(33) Björkman, T.; Gulans, A.; Krasheninnikov, A. V.; Nieminen, R. M. van der Waals Bonding in Layered Compounds from Advanced Density-Functional First-Principles Calculations. *Phys. Rev. Lett.* **2012**, *108*, 235502.

(34) Lynch, R. W.; Drickamer, H. G. Effect of High Pressure on the Lattice Parameters of Diamond, Graphite, and Hexagonal Boron Nitride. *J. Chem. Phys.* **1966**, *44*, 181-184.

(35) Hanfland, M.; Beister, H.; Syassen, K. Graphite under pressure: Equation of state and first-order Raman modes. *Phys. Rev. B* **1989**, *39*, 12598-12603.

(36) Zhao, Y. X.; Spain, I. L. X-ray diffraction data for graphite to 20 GPa. *Phys. Rev. B* **1989**, *40*, 993-997.

(37) Solozhenko, V. L.; Will, G.; Elf, F. Isothermal compression of hexagonal graphite-like boron nitride up to 12 GPa. *Solid State Commun.* **1995**, *96*, 1-3.

(38) Zhao, Y.; Von Dreele, R. B.; Weidner, D. J.; Schiferl, D. P- V- T Data of hexagonal boron nitride h BN and determination of pressure and temperature using thermoelastic equations of state of multiple phases. *High Pressure Res.* **1997**, *15*, 369-386.

(39) Fuchizaki, K.; Nakamichi, T.; Saitoh, H.; Katayama, Y. Equation of state of hexagonal boron nitride. *Solid State Commun.* **2008**, *148*, 390-394.

(40) Clark, S. M.; Jeon, K.-J.; Chen, J.-Y.; Yoo, C.-S. Few-layer graphene under high pressure: Raman and X-ray diffraction studies. *Solid State Commun.* **2013**, *154*, 15-18.

(41) Birch, F. Finite Elastic Strain of Cubic Crystals. *Physical Review* **1947**, *71*, 809-824.

(42) Birch, F. Elasticity and constitution of the Earth's interior. **1952**, *57*, 227-286.

(43) Vinet, P.; Ferrante, J.; Smith, J. R.; Rose, J. H. A universal equation of state for solids. *Journal of Physics*

C: Solid State Physics **1986**, *19*, L467-L473.

(44) Vinet, P.; Smith, J. R.; Ferrante, J.; Rose, J. H. Temperature effects on the universal equation of state of solids. *Phys. Rev. B* **1987**, *35*, 1945-1953.

(45) Kolmogorov, A. N.; Crespi, V. H. Registry-dependent interlayer potential for graphitic systems. *Phys. Rev. B* **2005**, *71*, 235415.

(46) Godec, Y. L.; Martinez-Garcia, D.; Mezouar, M.; Syfosse, G.; Itié, J. P.; Besson, J. M. Thermoelastic behaviour of hexagonal graphite-like boron nitride. *High Pressure Res.* **2000**, *17*, 35-46.

(47) Wirtz, L.; Rubio, A. The phonon dispersion of graphite revisited. *Solid State Commun.* **2004**, *131*, 141-152.

(48) Serrano, J.; Bosak, A.; Arenal, R.; Krisch, M.; Watanabe, K.; Taniguchi, T.; Kanda, H.; Rubio, A.; Wirtz, L. Vibrational Properties of Hexagonal Boron Nitride: Inelastic X-Ray Scattering and Ab Initio Calculations. *Phys. Rev. Lett.* **2007**, *98*, 095503.

(49) Mandelli, D.; Ouyang, W.; Urbakh, M.; Hod, O. The Princess and the Nanoscale Pea: Long-Range Penetration of Surface Distortions into Layered Materials Stacks. *ACS Nano* **2019**.

(50) Ouyang, W.; Qin, H.; Urbakh, M.; Hod, O. Controllable Thermal Conductivity in Twisted Homogeneous Interfaces of Graphene and Hexagonal Boron Nitride. *Nano Lett.* **2020**, *20*, 7513-7518.
